# Structure of a human cap-dependent 48S translation pre-initiation complex

**DOI:** 10.1093/nar/gky054

**Published:** 2018-02-01

**Authors:** Boris Eliseev, Lahari Yeramala, Alexander Leitner, Manikandan Karuppasamy, Etienne Raimondeau, Karine Huard, Elena Alkalaeva, Ruedi Aebersold, Christiane Schaffitzel

**Affiliations:** 1 European Molecular Biology Laboratory, Grenoble Outstation, 71 Avenue des Martyrs, 38042 Grenoble, France; 2 ETH Zürich, Institute of Molecular Systems Biology, Auguste-Piccard-Hof 1, 8093 Zürich, Switzerland; 3 Engelhardt Institute of Molecular Biology, the Russian Academy of Sciences, 119991 Moscow, Russia; 4 Faculty of Science, University of Zürich, 8057 Zürich, Switzerland; 5 School of Biochemistry, University of Bristol, Bristol BS8 1TD, UK

## Abstract

Eukaryotic translation initiation is tightly regulated, requiring a set of conserved initiation factors (eIFs). Translation of a capped mRNA depends on the trimeric eIF4F complex and eIF4B to load the mRNA onto the 43S pre-initiation complex comprising 40S and initiation factors 1, 1A, 2, 3 and 5 as well as initiator-tRNA. Binding of the mRNA is followed by mRNA scanning in the 48S pre-initiation complex, until a start codon is recognised. Here, we use a reconstituted system to prepare human 48S complexes assembled on capped mRNA in the presence of eIF4B and eIF4F. The highly purified h-48S complexes are used for cross-linking/mass spectrometry, revealing the protein interaction network in this complex. We report the electron cryo-microscopy structure of the h-48S complex at 6.3 Å resolution. While the majority of eIF4B and eIF4F appear to be flexible with respect to the ribosome, additional density is detected at the entrance of the 40S mRNA channel which we attribute to the RNA-recognition motif of eIF4B. The eight core subunits of eIF3 are bound at the 40S solvent-exposed side, as well as the subunits eIF3d, eIF3b and eIF3i. elF2 and initiator-tRNA bound to the start codon are present at the 40S intersubunit side. This cryo-EM structure represents a molecular snap-shot revealing the h-48S complex following start codon recognition.

## INTRODUCTION

During eukaryotic translation initiation, 80S ribosomes are assembled on the messenger RNA. This process is highly regulated and can be divided into several phases: a ternary complex (TC) - consisting of trimeric eukaryotic initiation factor 2 (eIF2), GTP and initiator methionyl-tRNA (Met-tRNAi) - binds to the small ribosomal subunit (40S) to form the 43S pre-initiation complex (43S pre-IC). Subsequently, this 43S pre-IC complex attaches to the 5′-end of the mRNA which contains a cap, forming the 48S (pre-)initiation complex (48S). The 48S complex scans mRNA from 5′ to 3′ until it identifies an AUG start codon in an appropriate sequence context (Kozak consensus). Start codon recognition by the TC triggers phosphate release from eIF2 (1) followed by dissociation of eIF2•GDP from the 48S complex. Finally, eIF5B binds the 48S complex and mediates 60S subunit joining to form the elongation-competent 80S initiation complex (2,3).

All stages of translation initiation are highly coordinated and require a set of eukaryotic initiation factors (eIFs). In humans, canonical translation initiation on a 5′-capped mRNA involves factors eIF1, eIF1A, eIF2, eIF3, eIF4B, eIF4F, eIF5, eIF5B and Poly(A)-binding protein (2). eIF1 is thought to control the codon–anticodon interaction (2,4), while eIF1A stimulates the binding of the TC (eIF2•GTP•Met-tRNAi) to the 40S subunit and supports the formation of the codon–anticodon interaction (2–4). Primary functions of eIF2 are the selection and recruitment of Met-tRNAi to the 40S ribosomal subunit as well as controlling start site recognition (5). The largest and most complex of the initiation factors is eIF3, a ∼800 kDa complex. It comprises 12 subunits (a-m) in humans, and eIF3j which is loosely associated (6,7). eIF3 is essential during all stages of eukaryotic translation initiation (3,5). It binds the 40S subunit as well as eIF1 and eIF4G. It stimulates incorporation of the TC into the initiation complex and promotes attachment of 43S pre-IC complexes to mRNA and subsequent scanning (2,5). Yeast eIF3 has been shown to interact with eIF5 (8). In addition, eIF3 also possesses ribosome dissociation and anti-association activities, preventing untimely joining of the 40S and 60S subunits (9). eIF3 interacts with the mRNA cap-binding multiprotein complex eIF4F which is composed of the DEAD-box helicase eIF4A, the cap-binding subunit eIF4E and the scaffold protein eIF4G which mediates protein-protein as well as protein-mRNA interactions. The initiation factor eIF4B is required for the efficient recruitment of mRNA to the initiation complexes as shown in yeast, and it interacts with the helicase eIF4A which unwinds mRNA during the scanning process (10–12). eIF5 stimulates GTP hydrolysis by eIF2, but is required neither for 48S formation nor for correct start codon recognition (13). eIF5B mediates joining of 60S and the dissociation of eIF2•GDP from the 48S complex (3).

Several structures of eukaryotic initiation complexes were determined by electron cryo-microscopy (cryo-EM) providing essential mechanistic insights into the molecular events and conformational changes in the ribosomal complexes during mRNA binding and start codon recognition. Structures were determined of a mammalian 43S pre-IC without mRNA (14,15), yeast 40S–eIF1–eIF3 and 40S–eIF1–eIF1A–eIF3 complexes (16,17), as well as a partial yeast 48S pre-ICs lacking eIF4F and eIF4B with uncapped mRNA, visualizing important interactions between eIF1, eIF1A, eIF2, eIF3 and the 40S ribosomal subunit (4,18). More recently, the cryo-EM structure of a ribosomal post-splitting complex revealed that the recycling factor ABCE1 is part of the 43S pre-initiation complex (19). This led to a revision of the interpretation of the late-stage 48S pre-IC purified from rabbit reticulocyte lysate (20,21): EM density at the 40S intersubunit side, close to the GTPase-binding site, which previously was attributed to subunits g and i of eIF3, now is attributed to ABCE1 which was co-purified from the cell lysate (21). Taken together, this led to the proposal that the recycling factor ABCE1 could be part of 48S pre-IC, with putative roles in anti-association of ribosomal subunits and stabilization of initiation factor binding (21).

Here, we reconstituted the human 48S pre-initiation complex (h-48S) from purified factors on capped mRNA in the presence of eIF4F and eIF4B and a non-hydrolysable GTP analogue (GMPPNP). Affinity purification of the h-48S complex led to an unprecedented enrichment of functional initiation complexes enabling biochemical and structural characterization. We solved the structure of the h-48S complex at a resolution of 6.3 Å by cryo-EM. The TC (eIF2•GMPNP•Met-tRNAi) makes codon–anticodon contacts in the h-48S structure. We find the eIF3 PCI/MPN core and the so-called Yeast-Like-Core (YLC) subcomplex (C-terminal part of eIF3a, eIF3b and eIF3i) (6) bound to the solvent-exposed side in the h-48S IC structure. The subunit eIF3d was described as a cap-binding factor essential for assembly of initiation complexes on specific mRNAs (22). The crystal structure of eIF3d (22) fits unambiguously into the cryo-EM reconstruction at the exit of the mRNA channel, but does not contact the mRNA. At the entrance of the mRNA channel, additional density is attributed to the RNA-recognition motif (RRM) of eIF4B, ideally positioned to keep the mRNA unwound. We corroborate the location of eIF4B and of eIF3 subunits in the h-48S complex by cross-linking/mass spectrometry (XL-MS) analysis.

## MATERIALS AND METHODS

### Plasmids

The plasmid pET28a-MVHL-STOP2 is derived from pET28a-MVHL-STOP (23). It contains four CAA repeats followed by 52 nucleotides β-globin 5′-UTR, the coding region for Met, Val, His, Leu (MVHL), the stop codon UAA, the rest of the β-globin ORF followed by downstream sequences complementary to a DNA oligonucleotide for RNaseH cleavage and a biotinylated oligonucleotide for affinity purification (Supplementary Figure S1A). Plasmids for expression of eIF1, eIF1A, eIF4A, and eIF5 are described in (13,24). The gene encoding human eIF4B was sub-cloned from the plasmid pET(His6-eIF4B) (24) into the pACEBac1 vector via *Rsr*II and *Sal*I restriction sites for insect cell expression.

### *In vitro* transcription and mRNA capping

The plasmid pET28a-MVHL-STOP2 was amplified by PCR using specific oligonucleotides (forward primer 5′-TCCGGCGTAGAGGATCGAGATC-3′, reverse primer 5′-GACTCGAGCAGATCTATTAAGAGCGGTCGGTAAAACTTCGGCCAGTGAATTTCAGTGGTATTTGTG-3′). The PCR product was transcribed *in vitro* using T7 RNA polymerase and purified by LiCl/EtOH precipitations. The purified mRNA was capped using Vaccinia Virus Capping Enzyme and the ScriptCap m7G Capping System (CellScript).

### Human initiation factors, 40S ribosome purification and tRNA aminoacylation

Native human factors eIF2, eIF3, eIF4F and human 40S ribosomal subunits were purified from *HeLa* cytoplasmic lysate (Cilbiotech SA, Belgium) as described in (23). Human eIF1, eIF1A, eIF4A, and eIF5 were expressed as recombinant proteins in *Escherichia coli* and purified as described (23,25). His-tagged human eIF4B was expressed using the Multibac/insect cell expression system (26). For eIF4B purification, insect cell pellets from ∼1 l of culture were dissolved in the protein buffer L (20 mM Tris–HCl, pH 7.5, 100 mM KOAc, 5% glycerol, 2 mM DTT). The cells were lysed by four freeze-thaw cycles. Subsequently, the cleared cell lysate was applied onto a HisTrap column (GE Healthcare) equilibrated with buffer L. After washing with buffer L supplemented with 10 mM imidazole, eIF4B was eluted by an imidazole gradient from 10 mm to 200 mM in buffer L. The fractions containing eIF4B were applied onto a MonoQ column (GE Healthcare) equilibrated with buffer L. eIF4B was eluted by a KCl gradient from 100 to 500 mM in buffer L. Met-tRNAi was amino-acylated using recombinant *E. coli* methionyl-tRNA synthetase (23).

### 48S complex preparation

Human 48S complexes were assembled as described in Ref. (23) with the following modifications: The 48S assembly was performed in reaction buffer A (20 mM Tris–HCl, pH 7.5, 50 mM KOAc, 2.5 mM MgCl_2_, 2 mM DTT, 0.25 mM spermidine) supplemented with 200 U RiboLock RNase inhibitor (Thermo), 1 mM ATP, 0.2 mM GMPPNP, 35 pmol of capped MVHL-stop mRNA, 35 pmol Met-tRNAi, 50 pmol purified human small ribosomal subunits (h-40S), 100 pmol eIF2, 50 pmol eIF3, 80 pmol eIF4F, eIF4A, eIF4B, eIF1, eIF1A, eIF5 each, in a volume of 500 μl. The reaction mix was incubated for 30 min at 37°C. Subsequently, a biotinylated 2′-*O*-methyl-RNA oligonucleotide (IBA GmbH) with the sequence 5′-dT*dT*dT*CAGAUCUAUUAA GAGCGGUCGGdT*dT*dT*-3′ and Streptavidin High-Capacity Agarose (Thermo) were used for affinity purification of the h-48S complexes. The streptavidin beads were washed in buffer B (20 mM Tris–HCl, pH 7.5, 40 mM KOAc, 7.5 mM MgCl_2_, 2 mM DTT, 0.25 mM spermidine) until the wash fractions were protein-free (based on OD_280 nm_ measurements). Subsequently, the oligonucleotide 5′-TTCGGCCAGTGAATTTC-3′ was allowed to anneal to the mRNA. The mRNA-bound complexes were eluted from the streptavidin beads by addition of 140U RNaseH (New England Biolabs) in reaction buffer A supplemented with 0.25 mM GMPPNP.

The ribosomal complexes were analysed by toe-print assays (primer extension inhibition) using AMV reverse transcriptase (Promega) and a 6-carboxyfluorescein (FAM)-labeled oligonucleotide (5′-GCAATGAAAATAAATTTCC-3′) complementary to the 3′-UTR region of the MVHL-stop mRNA. The resulting fragments were analyzed by commercial sequencing services (capillary sequencing, FASTERIS SA, Switzerland). Based on the intensity of toe-print signals, around one third of the h-48S complexes were retained after elution from the beads.

### Electron cryo-microscopy

Data were collected on a FEI Titan Krios microscope (MPI Göttingen) operated at 300 kV under low-dose conditions (30 ± 5 e^−^/Å^2^) using a defocus range of 1.5–4 μm. Single-frame images were recorded on a Falcon II detector at a calibrated magnification of 112,000 (yielding a pixel size of ∼1.25 Å). We discarded micrographs that showed noticeable signs of astigmatism or drift. 2,380 micrographs were used for image processing.

### Image processing

192,543 particles were picked semi-automatically using EMAN2 (27). The parameters for the contrast transfer function were estimated for the micrographs using CTFFIND4 (28). 2D class averaging, 3D classifications, and refinements were performed with RELION (29). After 2D class averaging, classes with aberrant particles were discarded. 165,372 particles belonging to the best 2D classes were selected for 3D reconstruction. To obtain an initial reconstruction, the 40S + HCV IRES cryo-EM reconstruction (EMD-3019) (30) was low-pass filtered to 60 Å and used as an input model. Subsequently, all selected particles were classified into four classes by 3D classification. Two of the four classes could be refined to high resolution: Class 1 (30.6% particles; 6.3 Å; h-48S complex) and Class 3 (42.5% particles; 6.6 Å; 40S with TC). Class 2 (5.5% particles) contained dimers of 40S + eIF3. Class 4 (21.4% particles) was similar to Class 1. The resolutions reported are after gold-standard refinement and using the FSC = 0.143 criterion (31). The local resolution was estimated using RESMAP (32). All maps were further processed for the modulation transfer function of the detector and sharpened by applying a negative B factors (–91 Å^2^ for the h-48S map and –165 Å^2^ for the 40S-TC map; estimated as in (33)).

### Fitting of atomic structures into the cryo-EM maps

Volumes obtained by RELION were segmented in Chimera using the SEGGER tool (34,35). The atomic models of 40S, tRNAi and eIF2 subunits from the mammalian 48S model (20), atomic models of different eIF3 subunits (15) as well as the crystal structure of *Nasonia vitripennis* eIF3d (22) and the NMR structure of the eIF4B RRM (36) were fitted into the h-48S map using Chimera. The figures were generated by PyMOL (The PyMOL Molecular Graphics System, Version 1.8 Schrödinger, LLC) and by Chimera (34).

### Cross-linking/mass spectrometry analysis

Affinity purified h-48S complexes were cross-linked with 1 mM DSS-d0/d12 (Creative Molecules) in buffer C (20 mM HEPES–KOH pH 8, 100 mM KCl, 5 mM MgCl_2_, 0.25 mM GMPPNP), dialyzed against water and lyophilized.

Processing of the cross-linked complexes, LC–MS analysis and data processing with xQuest was essentially performed as described in (37), with the exception that all MS data was acquired on a Thermo Orbitrap Fusion Lumos mass spectrometer connected to a Thermo Easy-nLC 1200 HPLC system. The Lumos instrument was operated in data-dependent top speed acquisition mode with a cycle time of 3 s and using collision-induced dissociation in the linear ion trap for fragmentation. One data set was acquired in high/low resolution mode with precursor ion detection in the Orbitrap analyser (resolution = 120 000) and fragment ion detection in the linear ion trap (rapid scan mode setting). A second data set was acquired in high/high resolution mode with both precursor and fragment ion detection in the Orbitrap (at 60 000 and 30 000 resolution, respectively).

MS/MS spectra were searched against a database containing all 40S ribosomal subunits, initiation factors and nine contaminant proteins (including keratins). xProphet (38) was used to adjust the false discovery rates (FDR) to <5% with the corresponding xQuest score cut-offs at 23.9 for inter-protein cross-links and 16.0 for intra-protein cross-links in high/low mode, and 30.0 for both inter- and intra-protein cross-links in high/high mode. Supplementary Table S1 includes all confident identifications and the scores as reported by xQuest. The closer the score to the threshold, the more likely it is to be a false positive.

The overlaps for the high/high and high/low experiments were as follows: calculated from unique peptide-pairs: 50.4% for intra-protein cross-links (70 out of 139; 31 unique to high/high, 38 unique to high/low) and 37.7% for inter-protein cross-links (26 out of 69; 34 unique to high/high, 9 unique to high/low). The lower overlap and the contribution of more unique cross-links by the high/high dataset for inter-protein cross-links can be explained by the stronger discriminatory power of high mass accuracy MS/MS data for the much larger search space. Absence of a cross-link in one dataset may also be explained by the fact that a cross-link was identified, but fell below the 5% FDR threshold.

## RESULTS

### Preparation of h-48S pre-initiation complexes

We prepared h-48S complexes using a reconstituted *in vitro* system which enables preparation and study of mammalian translation complexes in defined states (23). The mRNA template comprised a 5′-cap, four CAA repeats upstream of the β-globin 5′-UTR and a short open reading frame (ORF) encoding the tetrapeptide MVHL (Supplementary Figure S1A). The ORF is followed by an UAA stop codon, the remaining β-globin ORF as 3′-UTR and additional sequences at the 3′-end to facilitate affinity purification. The CAA repeats were included to minimize secondary structure formation in the 5′-UTR of the mRNA. The sequence at the 3′-end of the mRNA comprises two regions for primer annealing (Supplementary Figure S1A): one of the primers, a biotinylated 2′-*O*-methyl-RNA oligonucleotide, is added to anneal to the 3′-end of mRNA before the 48S assembly reaction. To trap the h-48S complexes and to avoid phosphate release by eIF2, which would lead to its dissociation, we performed the *in vitro* h-48S assembly in the presence of a non-hydrolysable GTP analogue (GMPPNP). The reaction was started by addition of human 40S subunits and initiation factors purified either from HeLa cytoplasmic extract (eIF2, eIF3, eIF4F) or produced recombinantly in *Escherichia coli* (eIF1, eIF1A, eIF5, eIF4A) or in insect cells (eIF4B) (Supplementary Figure S1B). After the h-48S assembly reaction, the mRNA was immobilized on streptavidin beads and washed. Subsequently, a DNA primer was added to anneal to the 3′-end of the mRNA (Supplementary Figure S1A), and RNaseH was used to cut the mRNA and to thereby elute the h-48S complexes from the beads.

Our purification procedure ensures that mRNA-bound ribosomal complexes are highly enriched. In SYPRO Ruby-stained SDS gels the larger initiation factor subunits (eIF4G, eIF3a,b,c,d,l,e,f,h,m, eIF4B, eIF5, eIF4A, eIF2α,β,γ) can be detected (Supplementary Figure S1B). Because eIF1 and eIF1A have the same molecular weight as the bulk of the 40S ribosomal proteins, their presence in the sample cannot be confirmed by SDS-PAGE. The formation of h-48S complexes at the start codon as well as their presence in the elution fractions was verified by primer extension assays. A characteristic toe-print peak which is located 15–17 nucleotides downstream of the AUG codon was detected in the input and the elution fractions showing that the 40S is positioned at the start codon (Supplementary Figure S1C). These h-48S complexes were used for XL-MS and cryo-EM.

### Overview of cryo-EM structures

We determined the structure of the h-48S complex by single particle cryo-EM. We collected a data set of 4,419 micrographs, picked particles semi-automatically and cleaned the data set by 2D classification to remove non-ribosomal particles (Supplementary Figure S2A and B). 165,372 particles were subjected to 3D classification, resulting in four classes (Supplementary Figure S2C). Class 1 presents the h-48S complex (corresponding to 30.6% of the data set), Class 2 comprises dimers of 40S with eIF3 (5.5%), Class 3 40S–eIF2–Met–tRNAi complexes (42.5%), and Class 4 lower-quality h-48S complexes which could not be further refined, likely comprising particles from thicker ice regions (21.4%). Taken together, more than half of the sample comprises h-48S complexes (Supplementary Figure S2C).

**Figure 1.**
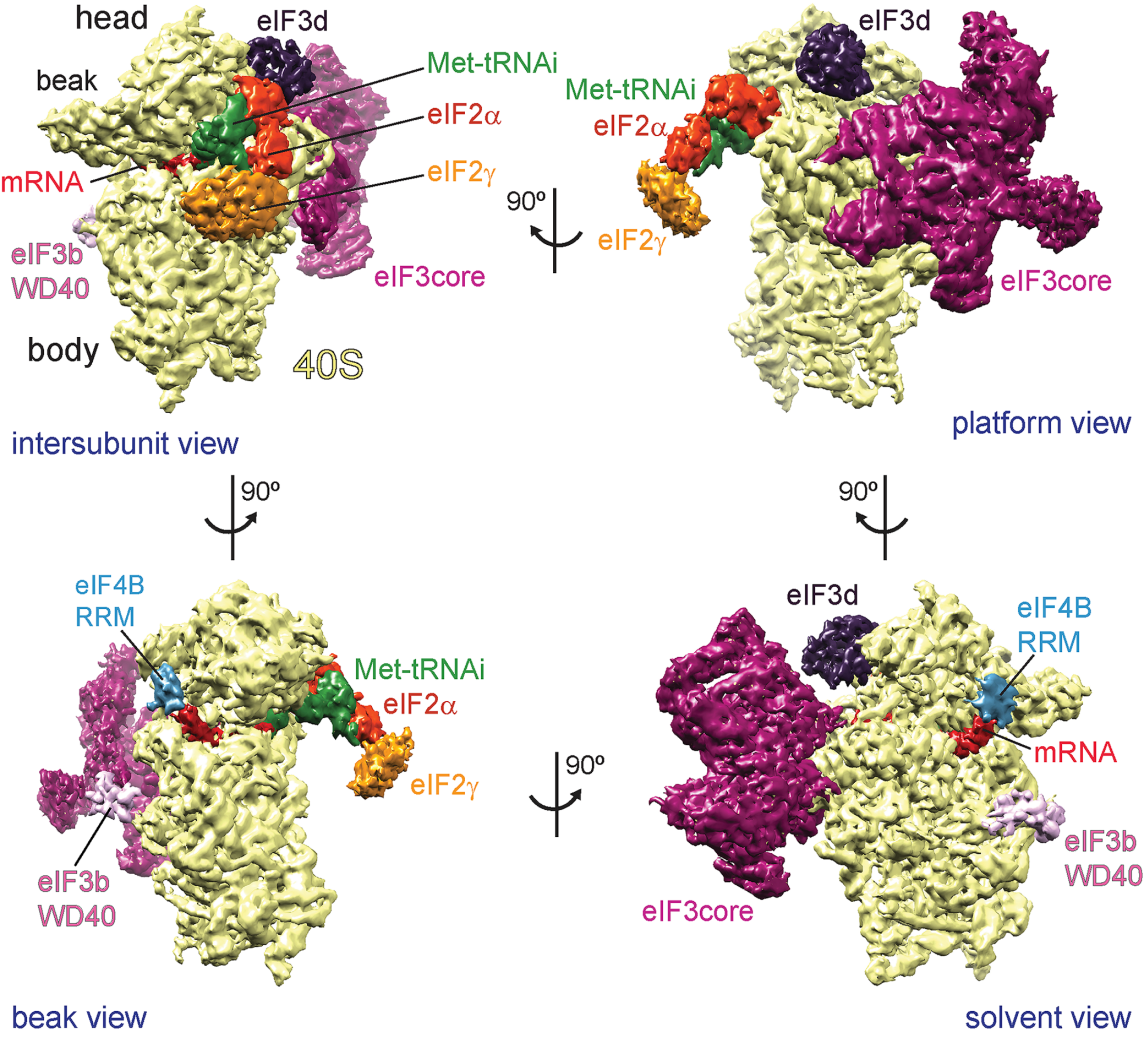
Segmented cryo-EM structure of the human 48S complex shown in four different views. The h-48S cryo-EM map was segmented for the 40S ribosomal subunit (yellow), the different initiation factors, initiator tRNA (Met-tRNAi, green) and mRNA (red). eIF2α is coloured in orange-red, eIF2γ in orange, the eIF3 PCI/MPN core in purple, eIF3d in dark purple, eIF3b subunit in light-pink and the eIF4B RRM in cyan. Arrows indicate the spatial relationship between the different views.

After refinement and post-processing of Class 1 in RELION (29), the h-48S complex reconstruction reached an overall resolution of 6.3 Å (Figure 1, Supplementary Figure S2C and D). The local resolution is highest for the 40S core and the factors directly contacting the 40S subunit (Supplementary Figure S2E). We segmented the h-48S map (Class 1) according to the cryo-EM structures of mammalian 43S pre-IC, 48S late-stage complexes and the yeast 48S pre-IC (4,15,20,21), showing similar density.

We further refined the volume corresponding to 40S–eIF2–Met–tRNAi complexes (Class 3) to an overall resolution of 6.6 Å (Supplementary Figure S2C and F). This volume closely resembles the h-48S structure (Class 1), but completely lacks densities assigned to eIF3 (Supplementary Figure S3). Importantly, eIF3 is indispensable for 48S formation on regular mRNAs such as the β-globin mRNA used in our study (3). Therefore, we assume that eIF3 was initially bound and dissociated during purification or grid preparation, resulting in 40S–eIF2–Met–tRNAi initiation complexes.

#### The human 48S complex

Similar to the 43S pre-IC structure (15), the structure of the h-48S complex (Class 1 and Class 4) displays high-resolution density for the eIF3 PCI/MPN core complex bound to the solvent-exposed side of the 40S subunit (Figure 1). At the same time, the 40S conformation and the binding of Met-tRNAi complexed with eIF2α and eIF2γ are virtually identical to the 48S late-stage structure which comprises ABCE1 but lacks eIF3 (20,21). This is in accordance with the fact that both complexes represent a state after start codon recognition. In the h-48S structure, the path of the mRNA through the mRNA channel formed by 40S can be followed (Figure 1, Supplementary Figure S4). The initiator tRNA (Met-tRNAi) is fully accommodated in the mRNA channel, and the tRNA anticodon loop contacts the start codon on the mRNA (Supplementary Figure S5B,C). Compared to the yeast pre-IC 48S structure (4), the TC in the h-48S complex moves towards the 40S head without affecting the codon–anticodon contacts and thus start codon recognition (Supplementary Figure S5).

Closer inspection of the TC density in the h-48S structure prior to refinement, revealed that additional, lower resolution density is present which we tentatively attribute to eIF2β (Supplementary Figure S6). This density is located next to Met-tRNAi, between eIF2γ and the 40S subunit. This tentative assignment is based on structural data of the archaeal homologues of eIF2 (39) and XL-MS experiments (see below) detecting cross-links between eIF2β and eIF2γ. Likely, eIF2β is flexibly bound to the TC since its EM density largely disappears during refinement.

**Figure 2.**
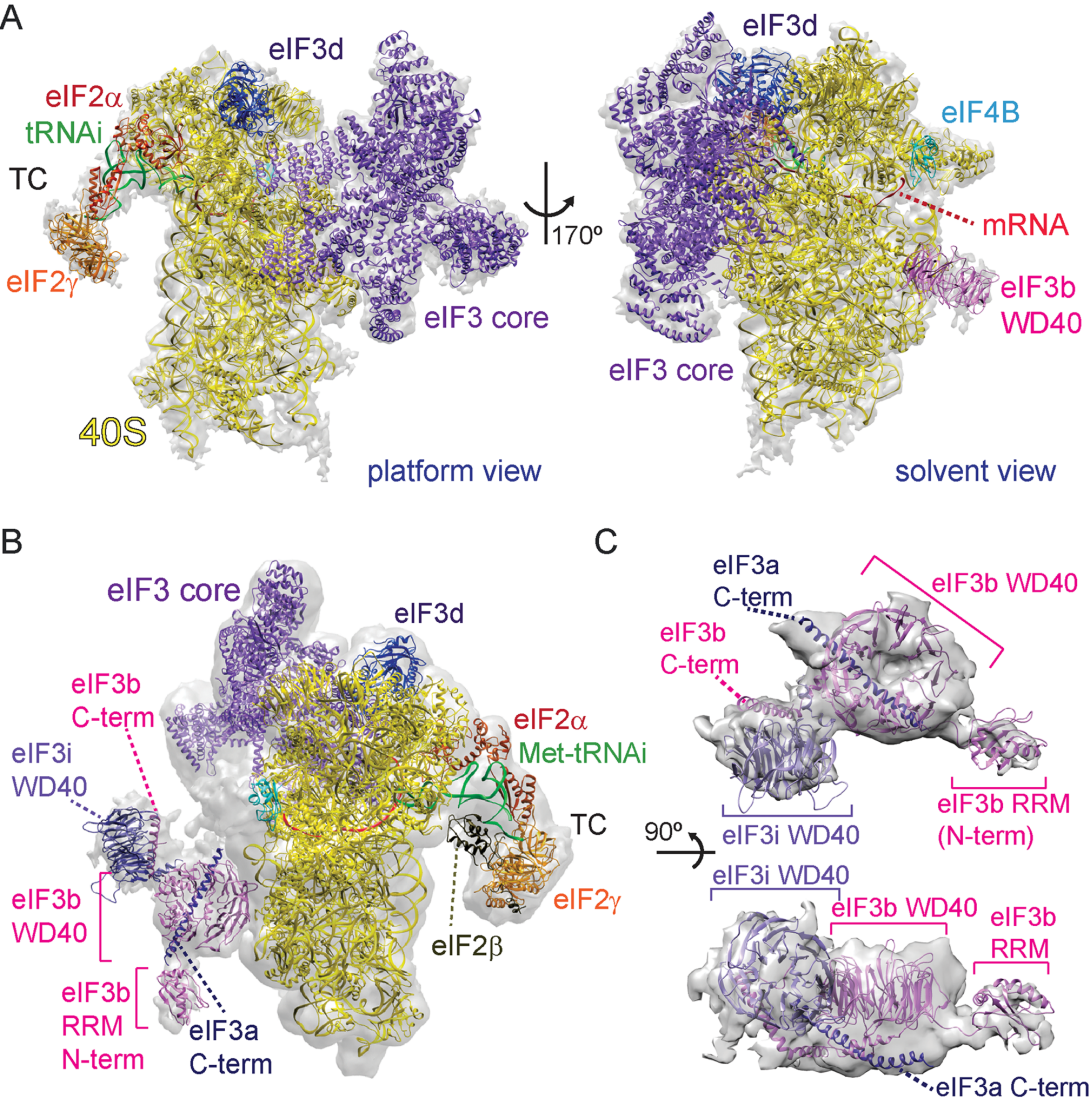
Initiation factor 3 core and YLC subunits are bound to the solvent-exposed side of 40S in the human 48S complex. (**A**) Fitting of the atomic structures of the eIF3 PCI/MPN core (purple), eIF3d (dark purple) and of the WD40 domain of eIF3b (pink) into the cryo-EM density of the human 48S complex (gray transparent). The fitted structure of eIF4B RRM is colored cyan, eIF2α orange-red, eIF2γ orange and Met-tRNAi (green). (**B**) In the h-48S structure prior to refinement the subunits eIF3b (pink), eIF3i (purple) and the C-terminal part of eIF3a (dark blue) are visible, as well as the β-subunit of eIF2 (olive) which is part of the ternary complex (TC). (**C**) Zoom to the YLC subunits eIF3b and eIF3i interacting with the C-terminus of eIF3a, shown in a top (above) and a side view (below).

The position of eIF3 in the h-48S structure resembles that in the mammalian 43S pre-IC structure (15): the eight eIF3 core subunits (a, c, e, f, h, l, k and m), eIF3d and the WD40 β-propeller domain of eIF3b are bound to the solvent-exposed side of 40S (Figures 1 and 2A). Notably, the h-48S map prior to refinement comprises additional, low-resolution density next to eIF3b. We attribute this density to the eIF3b RRM, the eIF3i WD40 domain and the C-terminal part of eIF3a (Figure 2B and C). This is corroborated by our XL-MS results (see below) and by the fact that density at the same position, of virtually identical size and shape was attributed to these subunit domains in the mammalian 43S pre-IC (15). In the h-48S structure, these domains seem to be rather flexible, however, as their density disappears during refinement. In comparison, the eIF3b WD40 domain was found close to eIF2γ and the eIF3b RRM next to eIF1 at the 40S intersubunit side in the yeast 48S pre-IC structure ((4) re-interpreted in (20)). In combination with our cryo-EM structure this suggests a relocation of eIF3b, most likely together with the other components of the YLC sub-complex eIF3i and eIF3g, during or immediately after start codon recognition from the intersubunit to the solvent-exposed side. As a result, only the TC remains bound to the 40S interface in the h-48S. Such a conformational change is compatible with subsequent initiation events, i.e. eIF2 dissociation and 60S subunit joining.

Notably, the h-48S reconstruction lacks densities for eIF1, eIF1A and eIF5. We were not able to confirm the presence of eIF1 in the purified h-48S sample. However, eIF1A and eIF5 are found in the h-48S preparations as evidenced by SDS-PAGE analysis (Supplementary Figure S1B) and XL-MS (see below): we could detect high-confidence cross-links between eIF1A and eIF2β and intra-molecular cross-links within eIF1A and eIF5 in the h-48S sample. As eIF1A participates in eIF5B recruitment and was shown to directly interact with eIF5B, eIF1A is expected to be part of the h-48S complex until the 60S subunit joins (40). We speculate that eIF1 could dissociate during purification and cryo-EM grid freezing procedures as it fulfilled its function in scanning and initiation site selection. Similarly, eIF1A and eIF5 could dissociate or be flexibly bound to the h-48S complex, and thus could not be detected in the cryo-EM reconstructions.

#### 40S–eIF3 dimers

Surprisingly, Class 2 comprises 40S dimers with two copies of eIF3, likely bound to mRNA (see preparation of h-48S complexes). The reconstruction was refined to 17 Å. The eIF3 PCI/MPN core and the subunits eIF3b and eIF3d are visible on both 40S subunits (Supplementary Figure S2C and G). Subunit eIF3d slightly moved towards the eIF3 PCI/MPN core, and the conformation of the 40S head is different compared to the h-48S structure (Class 1). The 40S dimerization is mediated by two contacts: the 40S body forms one contact, and the second contact involves the subunit interface of the 40S head and platform of both subunits. These contacts occlude the TC binding site on the 40S subunit interface (Supplementary Figure S2G). Notably, we did not identify monomeric 40S–eIF3 complexes without TC. Therefore, we conclude that these dimers were formed during *in vitro* 48S assembly. The formation of 40S–eIF3 dimers on β-globin mRNA has been reported previously, and binding of eIF3 to 40S was suggested to prevent 40S–60S re-association (41).

### Contacts of eIF3d with ribosomal proteins and 18S rRNA at the mRNA exit channel

**Figure 3.**
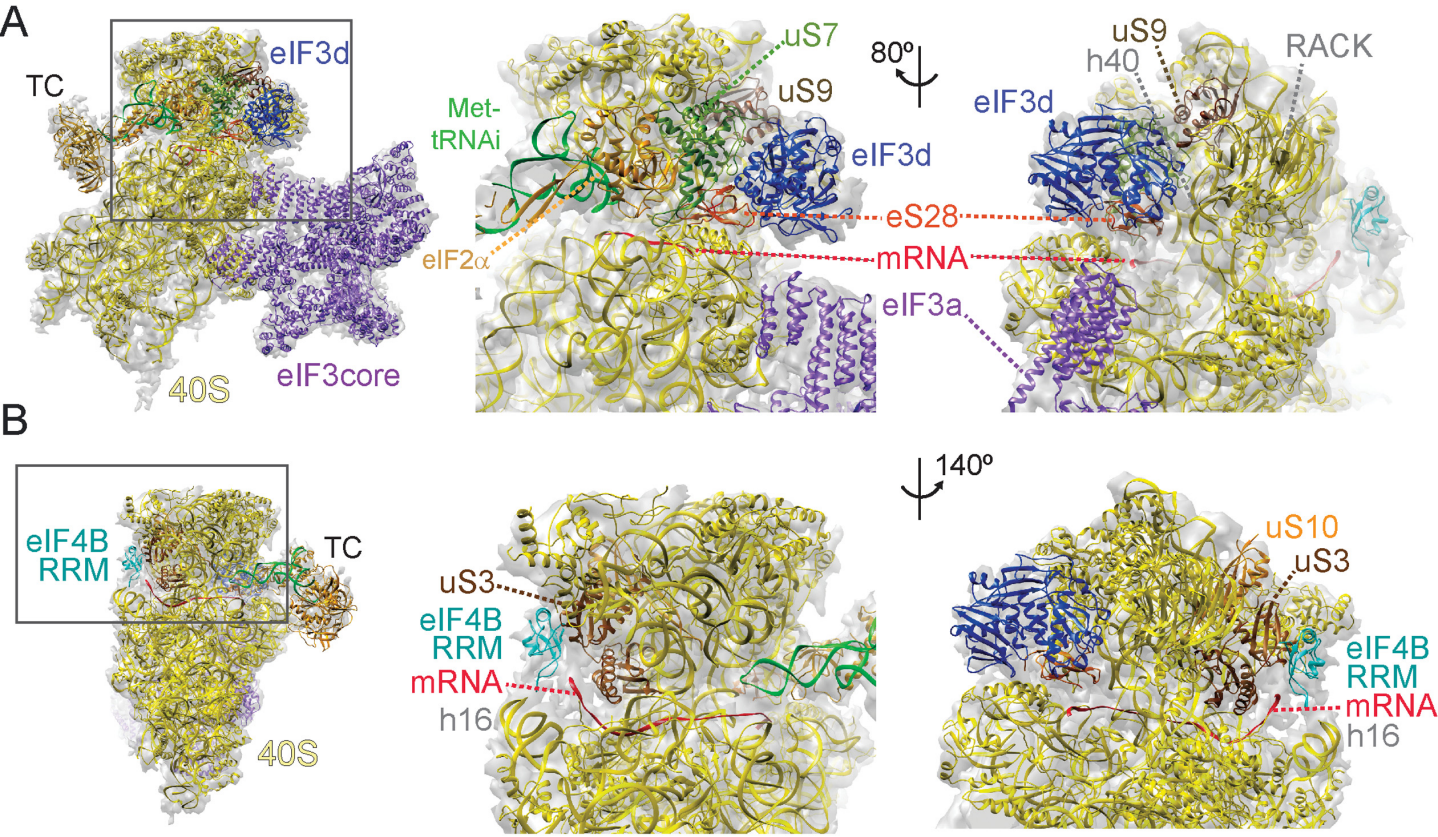
Factors eIF3d and eIF4B are bound at the mRNA exit and mRNA entry channel of 40S in the h-48S complex. (**A)** The subunit eIF3d is bound to the 40S head at the mRNA exit channel. The crystal structure of eIF3d (dark purple) (22) was fitted into the h-48S cryo-EM map. Close-up views (middle, right) of eIF3d binding next to ribosomal protein RACK1 and contacting uS7 (green), uS9 (brown), eS28 (orange) and helix h40 of 18S rRNA. (**B**) The NMR structure of the eIF4B RRM (34) (cyan) was fitted into the h-48S EM density at the entrance of the mRNA channel. Middle, right: close-up views of eIF4B binding in the vicinity of uS10 (orange). The eIF4B RRM contacts uS3 (brown) and helix h16 of 18S rRNA.

**Figure 4.**
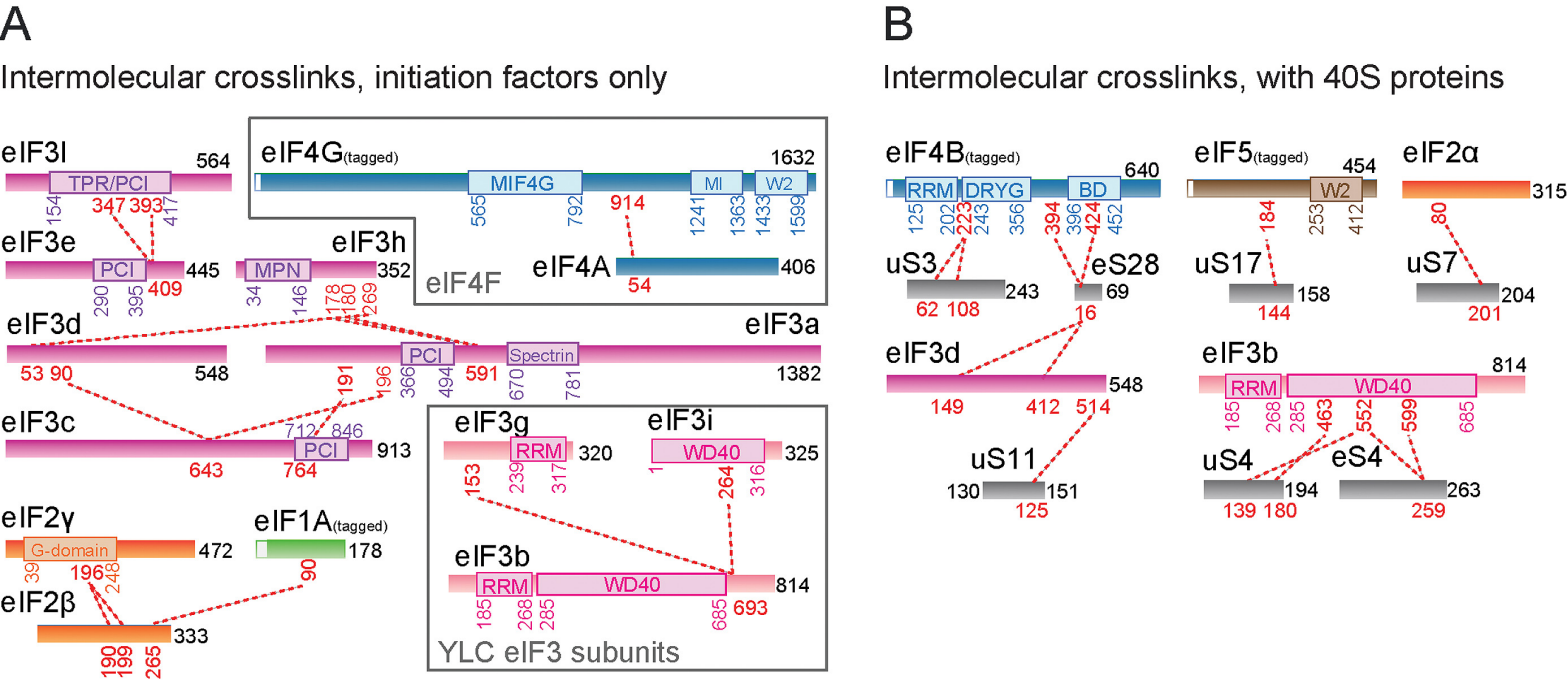
Cross-linking/mass spectrometry analysis of human 48S complex. (**A**) Cross-links identified between different eukaryotic initiation factors and their subunits are shown as red dashed lines. Cross-linked residues are indicated in red. Domain boundaries are indicated in the same colours as chosen for the protein. For clarity, only inter-molecular Lys-Lys cross-links by DSS are shown (intra-molecular cross-links and cross-links between ribosomal proteins are in Supplementary Table S1). Grey frames: subcomplexes of h-48S. (**B**) Cross-links between eIFs and 40S proteins. Nomenclature for ribosomal proteins adapted from Ref (42).

Above the mRNA exit channel, next to RACK1, we observe well-defined density (Figure 3A). This density was assigned to eIF3d in the rabbit 43S pre-IC (15). Therefore, we placed the recent crystal structure of eIF3d from the parasitoid wasp *Nasonia vitripennis* (22) into the corresponding density of the h-48S map. Chimera fitting indicates a cross-correlation value of 0.928 for the best fit of the eIF3d crystal structure into the corresponding density. We performed XL-MS to characterize the protein-protein interactions in the h-48S complex (Figure 4). For eIF3d, we obtained cross-links to ribosomal proteins uS11 and eS28 (nomenclature as in Ref. (42)). Our atomic model places eIF3d Lys412 within 12 Å cross-linking distance of eS28 Lys16 and eIF3d Lys514 within 20 Å distance to uS11 K125 (Figure 4, Supplementary Figure S7A) corroborating the position of eIF3d on the 40S head. In the h-48S structure eIF3d contacts uS7, uS9, eS28 and ribosomal RNA helix h40 (Figure 3A).

Recently, eIF3d was reported to possess 5′-cap-binding activity (22). This agrees with the position of eIF3d in the h-48S complex above the exit of the mRNA channel close to the 5′ end of the mRNA model. However, eIF3d was shown to bind only to specific mRNAs containing an eIF4F-inhibitory secondary structure element in their 5′-UTR, such as *c-Jun* mRNA. On these mRNAs, eIF3d-directed cap-dependent translation initiation can occur (22). The β-globin 5′-UTR does not contain such an element, and therefore, it is unlikely that eIF3d binds to its 5′-cap, in particular since eIF4F is present in the h-48S sample. Indeed, in the h-48S structure we do not observe extra density next to eIF3d that could be attributed to mRNA (Figure 3A).

### eIF4B is located at the mRNA channel entrance

After fitting 40S, eIF3 subunits, the TC and mRNA into the h-48S structure we observe additional density at the entry of the mRNA channel. Our attempts to improve the resolution of this density in this region of the map by focused classification and refinement (43) remained unsuccessful, probably due to the region of interest being small and yielding relatively poor signal.

The additional density in the h-48S map is found next to ribosomal protein uS3, close to uS10 and contacts rRNA helix 16 (Figure 3B). Previously, ribosomal protein uS10 was shown to interact with eIF4B in yeast using pulldown experiments (10). In the XL-MS analysis we identified cross-links between eIF4B Lys223 which is C-terminal to the eIF4B RRM and ribosomal protein uS3 residues Lys62 and Lys108 (Figure 4, Supplementary Figure S7B). Based on the cross-links as well as shape and size, we tentatively fitted the structure of the eIF4B RRM (36) into the corresponding density yielding a cross correlation value of 0.931 in Chimera (Figure 3B, Supplementary Figure S8).

We do not observe a direct interaction of uS10 with the eIF4B RRM. However, in the h-48S map prior to refinement (Class 1) we observe significant additional density next to the density we assigned to the eIF4B RRM (Supplementary Figure S7B) which we assume comprises other domains of eIF4B and possibly eIF4A. Interestingly, we also observe cross-links between eIF4B residues Lys394 and Lys424 and eS28 Lys16 (Figure 4). eS28 is located near the exit of the mRNA channel. The cross-links of eIF4B with uS3 and eS28 could indicate that eIF4B stretches along the 40S subunit, binds with its N-terminal RRM next to the mRNA channel entry and near the mRNA channel exit with its C-terminal basic domain (BD). However, eS28 is bound by eIF3d in the h-48S complex and eS28 Lys16 also cross-links to eIF3d (Figure 3A, Supplementary Figure S7A). In the structure, no additional unfilled density is observed next to eS28 and eIF3d (Supplementary Figure S7B), indicating that the eIF4B BD is rather flexible.

Notably, eIF3b also comprises a RRM. However, the WD40 domain of eIF3b binds to the surface of uS4 at the solvent-exposed side of the 40S subunit (44) (Figure 1). This positioning is further supported by cross-links between ribosomal proteins uS4, eS4 and the eIF3b WD40 domain (Figure 4B). Accordingly and in agreement with the cryo-EM structures of a yeast partial pre-IC complex (40S–eIF1–eIF1A–eIF3) (17) and of the 43S pre-IC (15), the RRM of eIF3b is then positioned close to the long helix of the C-terminal part of eIF3a and not at the entrance of the mRNA channel (Figure 2).

Our cryo-EM structure does not allow to model a sufficiently long portion of the mRNA to determine the RNA-binding mode of the eIF4B RRM. However, Arg135 and Lys137 are close to the mRNA in our model (Supplementary Figure S8B-D). These residues are conserved and known to be required for single-stranded RNA binding of eIF4B ((10) and references therein).

Interestingly, the DExH protein DHX29, which is required for scanning of structured mRNAs, binds to the same region of the 40S subunit in the mammalian 43S complex structure (14). Thus, it is tempting to speculate that this region could serve as a general docking site for RNA helicase complexes on the ribosome during translation initiation.

Based on hydroxyl radical foot-printing experiments, the eIF4B-uS10 interaction was suggested to induce conformational changes at the entrance of the mRNA channel to facilitate loading of the mRNA (10). The authors proposed that these conformational rearrangements involve ribosomal RNA helix 34. When we compared the conformation of h34 in the 43S pre-IC structure without eIF4B and mRNA (15) with its conformation in the h-48S structure (Supplementary Figure S9), we could not identify a significant conformational change. Therefore, we suggest that eIF4B stabilizes mRNA binding in the h-48S complex by directly contacting the mRNA and the 40S.

### Cross-linking/mass spectrometry of the h-48S initiation complex

To further characterize the h-48S complex and identify protein-protein interactions involving the initiation factors, we performed XL-MS experiments using the amine-reactive reagent, disuccinimidyl suberate (DSS) (Figure 4 and Supplementary Table S1). We find that the subunits of eIF3 forming the PCI/MPN core (eIF3a, eIF3c, eIF3e, eIF3h, eIF3l) cross-link with each other and with eIF3d which is in proximity (Figures 4A and 3A). Similarly and in agreement with previous results (15), YLC subunits eIF3b, eIF3i and eIF3g cross-link with each other, indicating that they form a stable subcomplex. No cross-links were detected between the eIF3 PCI/MPN core and the YLC subunits (Figure 4A). Taken together, this supports the concept that the eIF3 YLC subunits are linked flexibly to the PCI/MPN core (45) and indicates that these two apparently independent eIF3 parts do not form extensive contacts in the h-48S complex. XL-MS analysis corroborates the localization of eIF3b on the solvent-exposed side of 40S in the h-48S complex as observed in the cryo-EM structure (Figure 2): eIF3b cross-links with ribosomal proteins uS4 (44) and eS4 which both are located on the solvent-exposed side of 40S (Figure 4B).

Despite the fact that we could not identify density corresponding to eIF1A in the 48S structure, we obtained cross-links of eIF1A with the C-terminal domain of eIF2β (Figure 4A). This indicates that eIF1A is present in the h-48S complex. In agreement with these cross-links, in the partial yeast 48S pre-IC structure (lacking eIF3), eIF1A is positioned close to eIF2β (4). We assume that the association of eIF1A within the h-48S complex is very weak or the protein becomes flexible after mRNA scanning is completed, and therefore eIF1A is not visible in the cryo-EM reconstruction.

## DISCUSSION

The h-48S structure and XL-MS analysis reported here provide new insights into cap-dependent translation initiation, as the sample was prepared in the presence of capped mRNA, eIF4F, eIF4A and eIF4B. We report an optimized sample preparation protocol, resulting in high enrichment of 48S pre-initiation complexes. In fact, ∼52% of the ribosomal particles are h-48S complexes according to 3D classification (Classes 1 and 4). In addition to Coomassie-stained SDS PAGE and XL-MS experiments showing the protein content of our sample, we probed the position of the h-48S complex on the mRNA by toe-print (Supplementary Figure S1). We obtained the characteristic +15, +16, +17 peaks corresponding to the h-48S sitting on the AUG start codon of the β-globin mRNA (Supplementary Figure S1C).

Several functions have been attributed to eIF4B in translation initiation. Specifically, eIF4B is known to promote mRNA recruitment to initiation complexes (10), to stimulate the helicase activity of eIF4A (46) by increasing its processivity (47) and to stimulate eIF4A ATPase activity (48,49). Moreover, eIF4B has been shown to bind both mRNA and 40S (50). Interestingly, eIF4B is one of the targets of the PI3K-mTOR-S6K signalling pathway: phosphorylation of eIF4B by S6 kinase or protein kinase B leads to a stimulation of protein synthesis *in vivo* (51).

Supported by cross-links we observed, we suggest that the additional density we detect at the mRNA entry channel of the 40S corresponds to the RRM domain of eIF4B. At this position, eIF4B would be ideally situated to promote attachment of the 43S pre-IC to mRNA and to stimulate translation (10,50,51). eIF4B is known to form a stable complex with eIF4A and mRNA in presence of AMPPNP (11). In fact, we observe additional density around eIF4B in the unrefined h-48S volume (Supplementary Figure S7A), likely corresponding to more proteins than eIF4B alone which has a molecular weight of only 70 kDa. Therefore, we speculate that eIF4A is also located in this region interacting with eIF4B and the mRNA. In the 43S pre-IC structure, this region is occupied by DHX29 (14). It is unknown to date at which stage DHX29 dissociates from the ribosomal complexes. Consistently less DHX29 was detected in 48S compared to 43S complexes (52). This may indicate that DHX29 dissociates during 48S complex formation, possibly due to a competition with eIF4A which is part of the eIF4F complex and due to overlapping binding sites of DHX29 and eIF4A in the h-48S complex. By itself, eIF4A has an intrinsically low helicase activity which is strongly stimulated in the presence of eIF4B and eIF4G (53). The minimal processive unit for mRNA scanning consists of all three proteins: eIF4A, eIF4G and eIF4B (47), and all three factors are present in our sample as evidenced by MS (Figure 4). However, probably due to flexibility of the proteins involved, we can only resolve the RRM of eIF4B (Figure 3B).

Two models have been suggested addressing the mRNA binding to the 43S complex. In one model, the mRNA ‘slots’ directly into the open 40S mRNA channel (54). In the alternative model, the 5′end of the mRNA threads into the mRNA channel (54). Both models are compatible with our suggestion that eIF4B binds 40S at the mRNA entry channel and interacts with eIF4A to stimulate its ATP-dependent helicase activity. Single-stranded mRNA unwound by eIF4A can then be stabilized by eIF4B, facilitating the mRNA insertion via threading or slotting into the 40S mRNA channel.

It is interesting to note that we obtained a cross-link between eIF4A and eIF4G in the h-48S sample (Figure 4A). The cross-linked residues are in the N-terminal domain of eIF4A and in C-terminal part of the MIF4G domain of eIF4G. This finding agrees with contacts identified in the crystal structure of the complex of the MIF4G domain of eIF4G and a large part of eIF4A from *Saccharomyces cerevisiae* (55), suggesting that this interaction also occurs in the h-48S complex. However, eIF4A and eIF4G appear to be flexible in the h-48S complex, and we could not determine their position relative to 40S by EM or XL-MS. Multiple intra-molecular cross-links identified on eIF4G (Supplementary Table S1) provide additional evidence that this subunit is part of the h-48S complex as it contains multiple cross-linkable sites that are readily identified by XL-MS. In summary, eIF4G is part of the complex but interacts with the ribosome in a flexible manner. Factor eIF4E is the third component of the eIF4F complex. In contrast to eIF4A and eIF4G, we were unable to confirm its presence in the purified h-48S complex by XL-MS or cryo-EM. In fact, recent work suggests that the eIF4E subunit dissociates from the initiation complex when the mRNA is threaded into the mRNA channel (54) which would explain our finding.

In our sample, more than half of the particles comprise the eIF3 core, eIF3d, the YLC eIF3 subunits as well as the TC and eIF4B (Classes 1 and 4, Supplementary Figure S2C) and about 40% of our sample consists of 40S-eIF2/Met-tRNAi-mRNA complexes lacking eIF3 completely (Class 3, Supplementary Figure S2C). Since initiation on β-globin mRNA cannot occur in the absence of eIF3, we speculate that eIF3 may not be tightly associated at this late stage of translation initiation and can dissociate during sample preparation.

**Figure 5.**
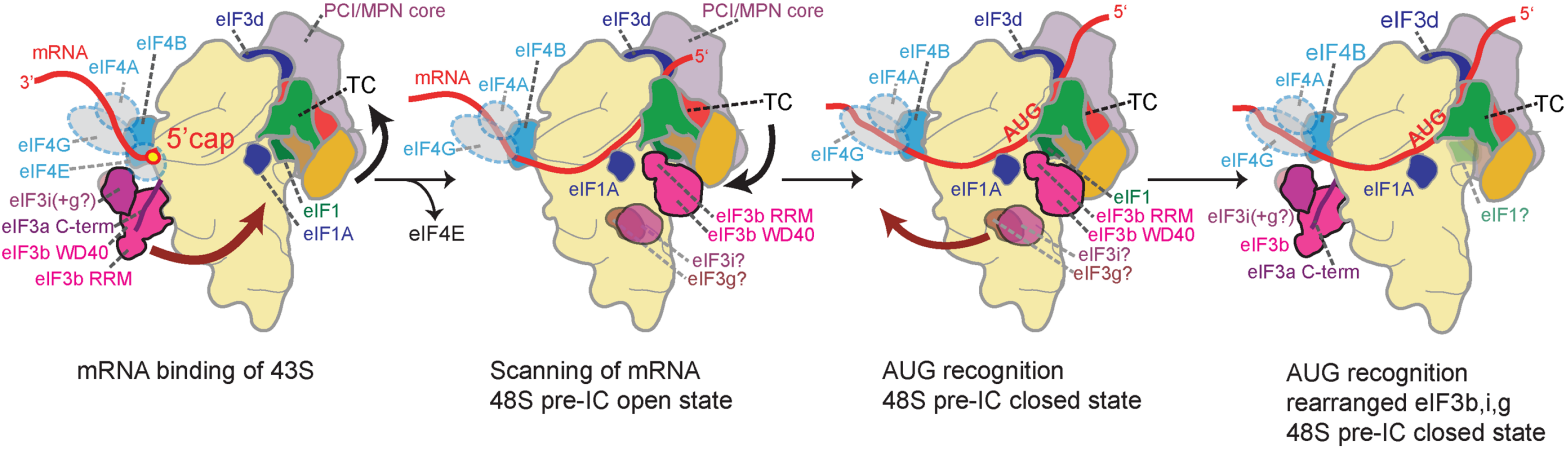
Model for h-48S conformational changes during 5′cap-dependent translation initiation. Left: 43S (comprising 40S-eIF3-eIF1-eIF1A, TC and eIF5) attachment to capped mRNA is supported by eIF4F (transparent blue) and eIF4B (cyan). Scanning (second panel) and AUG recognition (third panel) are supported by relocation of the eIF3b subunit, likely together with YLC subunits eIF3g and eIF3i, from the 40S solvent-exposed side to the intersubunit side (brown arrow). The black arrow indicates rearrangements of the TC during scanning and upon AUG recognition. Right: Start codon recognition results in conformational changes in the TC and re-location of the eIF3b subunit (likely together with eIF3g-eIF3i) back to the solvent-exposed side. Subsequent eIF2 dissociation from 48S (not shown) frees up the 40S interface for eIF5B-mediated 60S joining.

To reconcile these findings, we put forward the following model: The TC and eIF3 bind to the 40S subunit in the presence of eIF1, eIF1A and eIF5 forming the 43S pre-initiation complex. 43S pre-IC attachment to the 5′ region of capped mRNA bound to eIF4F and eIF4B leads to the 48S complex (Figure 5). If the mRNA is threaded into the 40S mRNA channel eIF4E likely dissociates from the 5′-cap (54). The 48S complex scans the mRNA from the first nucleotide of mRNA (54) which is only possible when the 5′-cap is in the mRNA channel and not associated with eIF4E. Genetic data indicate an important role of eIF3 and of the eIF4A–eIF4B–eIF4G complex in mRNA scanning (56,57).

The partial yeast 48S pre-IC complex structures ((4) and re-interpreted in (20)) suggest that the eIF3b subunit can move from the solvent-exposed side (where it is found in the 43S pre-IC (15)) to the intersubunit side of 40S during mRNA scanning until AUG recognition, thus promoting the movement of the 40S subunit on the mRNA (Figure 5). Considering that the interaction between the eIF3g–eIF3i subunits and eIF3b (mediated by the eIF3b C-terminus which is not visible in the cryo-EM structures) is highly conserved, it is likely that eIF3g and eIF3i relocate in complex with the eIF3b subunit from the solvent-exposed side to the intersubunit side (58). At the same time, the eIF4A–eIF4B–eIF4G complex which is located close to the mRNA entry channel can unwind the mRNA and stabilize mRNA-binding of 40S (Figure 5).

Upon start codon recognition, the TC undergoes a conformational change (Supplementary Figure S5, Figure 5). The eIF3b WD40 interacts with eIF2γ, and thus the TC conformational change could trigger the relocation of eIF3b (likely together with eIF3g-eIF3i) from the intersubunit side back to the solvent-exposed side where the YLC subunits are found in our h-48S pre-IC structure. Likely, eIF1A is flexibly bound to the 48S complex at this stage. eIF2γ has GTPase activity, and it has been shown that start codon recognition in the 48S complex triggers release of phosphate from eIF2 (1) which is stimulated by eIF5 and leads to dissociation of eIF2, leaving Met-tRNAi in the P-site of the 40S subunit. Finally, eIF5B-GTP promotes joining of the 60S subunit, forming an elongation-competent 80S initiation complex. eIF3 can remain bound to the solvent-exposed side of 40S, even after eIF2 release and after 60S subunit joining. Such a prolonged association of eIF3 with 40S has been demonstrated in yeast (59–61). In fact, re-initiation on mRNAs with short upstream open reading frames at a downstream start codon depends on the presence of eIF3 (3).

## DATA AVAILABILITY

The density maps and the atomic model of the h-48S complex have been submitted to the Electron Microscopy Data Bank (EMDB) and the Protein Data Bank (PDB) with accession codes EMD-4242, EMD-4265 and PDB ID 6FEC respectively. All other data supporting the findings of this study are available from the corresponding author upon request.

## Supplementary Material

Supplementary Data
