# Supplementary material for: Structure of a human cap-dependent 48S translation pre-initiation complex
Source: Nucleic Acids Res. 2018 Feb 1;46(5):2678–89. doi: 10.1093/nar/gky054 (PMC5861459; doi:10.1093/nar/gky054)
Supplement: Supplementary Data [file gky054_supp.zip › gky054_Supp.pdf]

Supplementary Figures

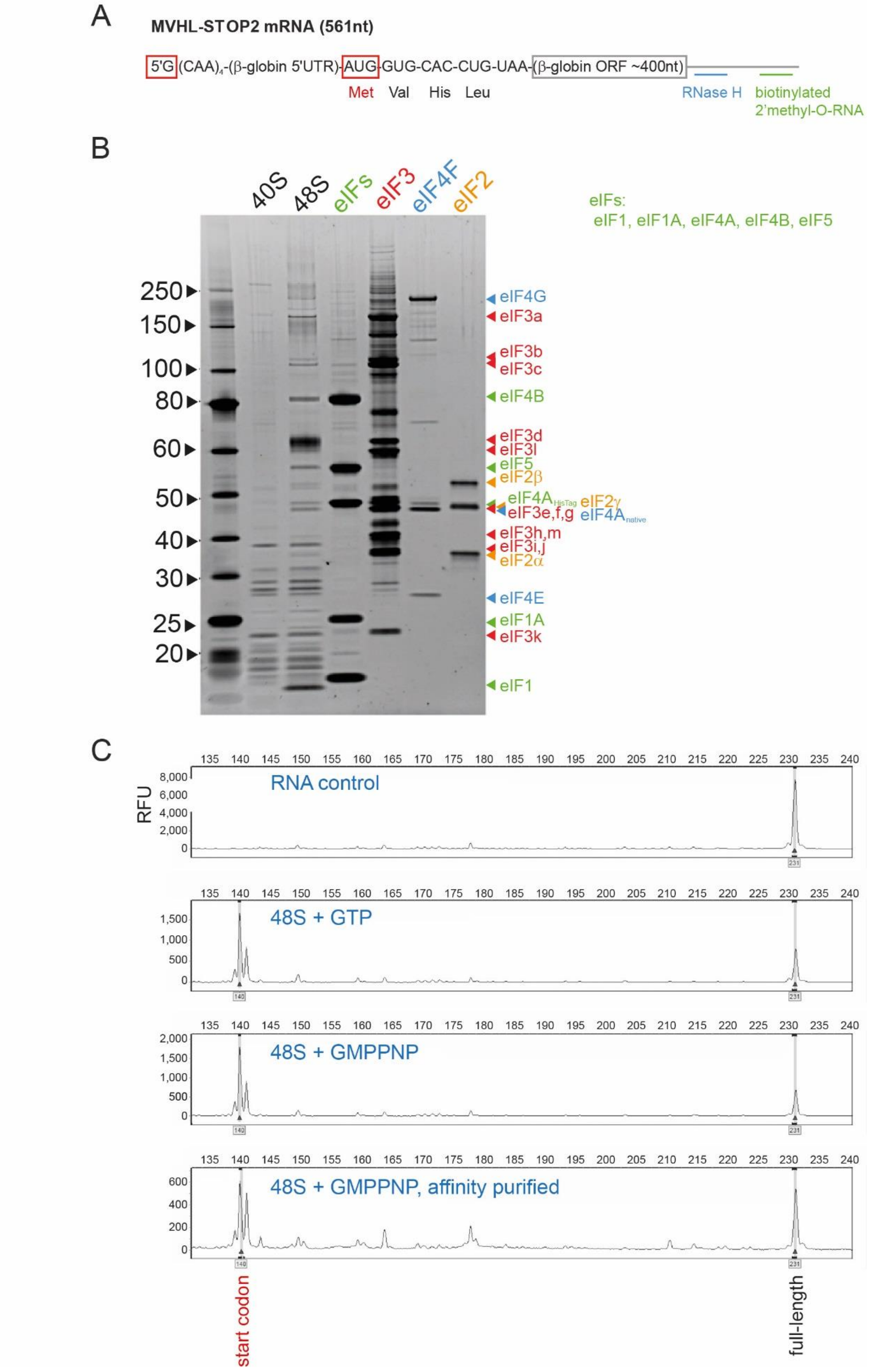

Supplementary Figure S1. Human 48S sample preparation. A. Scheme of MVHL-stop mRNA. The 5'-cap and the AUG start codon are shown in red boxes. Both UTRs are derived from the β-globin mRNA. The β-globin 3'-UTR is shown in a grey box; annealing sites for a DNA oligonucleotide for RNaseH cleavage and a biotinylated oligonucleotide for affinity purification (62) are indicated. **B.** SDS-PAGE analysis of the purified h-48S complex sample shown next to individual factors used for *in vitro* complex assembly. Initiation factors with a higher molecular weight than ribosomal proteins are readily identified in the h-48S sample. **C.** Toe-print analysis of h-48S complexes formed on MVHL-stop mRNA in the presence of GTP or its non-hydrolysable analogue GMPPNP. Ribosomal complexes bound to the mRNA inhibit cDNA synthesis by AMV reverse transcriptase leading to cDNA products of a specific length depending on the position of the ribosome on the mRNA. The DNA primer for reverse transcription is labelled at its 5' end with 6-carboxyfluorescein. Here, examples of the raw data obtained from capillary electrophoresis and fragment analysis are shown. H-48S complexes formed on β-globin mRNA are known to yield a characteristic toe-print corresponding to a position which is 16 nucleotides downstream of the adenine of the AUG start codon (63). A peak at ~140 nucleotides is expected on our model mRNA based on the distance between the adenine of the start codon and the 5' end of the primer, subtracting 15-17 nucleotides which are protected by the ribosome. After affinity purification (lowest panel) the sample is more diluted than in the assembly reaction; we estimate that ~1/3 of the ribosomal complexes are recovered. Accordingly, the background signals from the mRNA, which are present in all toe-print experiments, are more pronounced after purification.

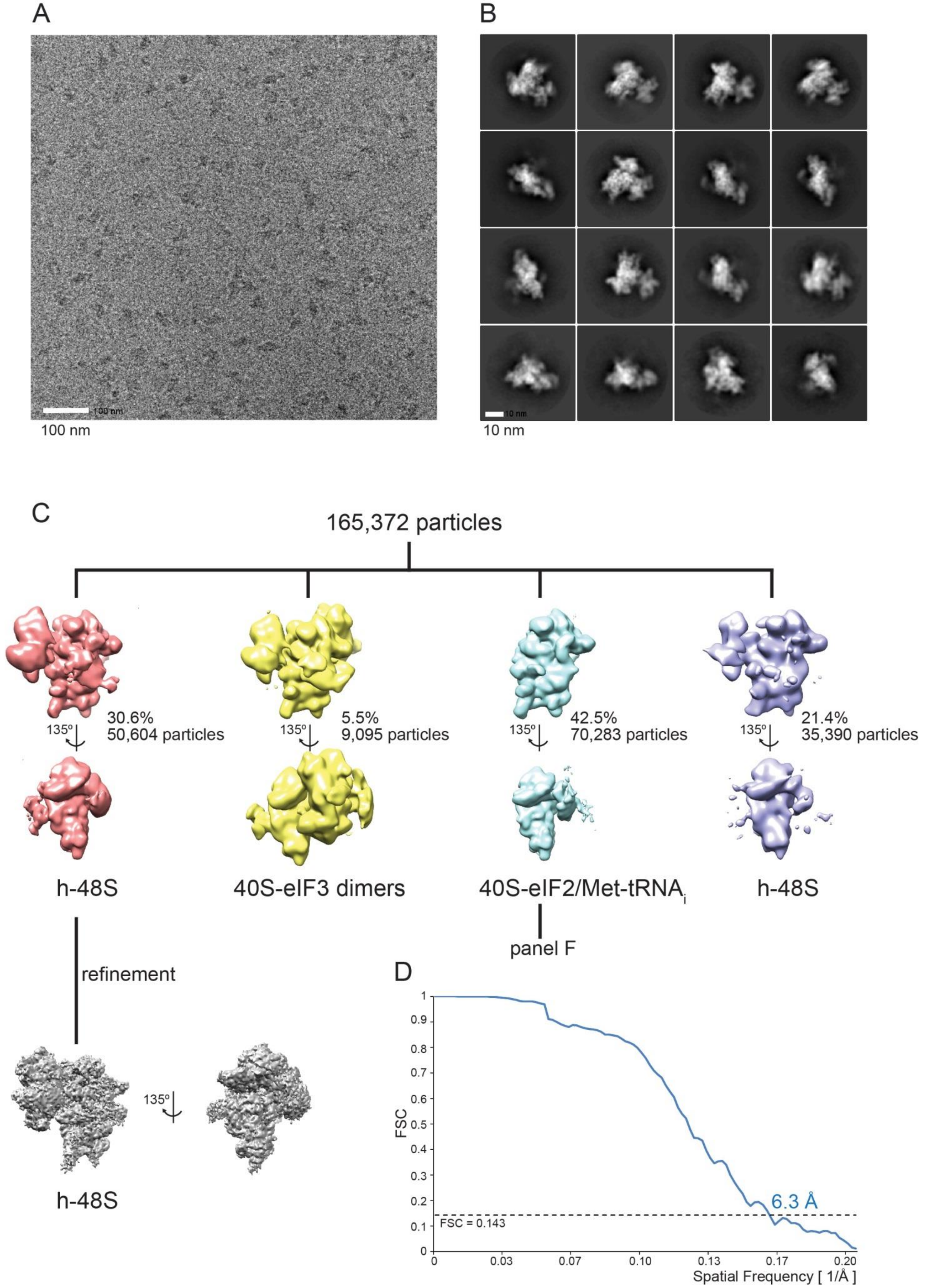
A
100 nm
B
10 nm
C
165,372 particles
30.6%
50,604 particles
5.5%
9,095 particles
42.5%
70,283 particles
21.4%
35,390 particles
135º
h-48S
40S-eIF3 dimers
40S-eIF2/Met-tRNAi
h-48S
panel F
refinement
h-48S
D
FSC
6.3 Å
FSC = 0.143
Spatial Frequency [1/Å]
0
0.03
0.07
0.10
0.13
0.17
0.20
0.1
0.2
0.3
0.4
0.5
0.6
0.7
0.8
0.9
1

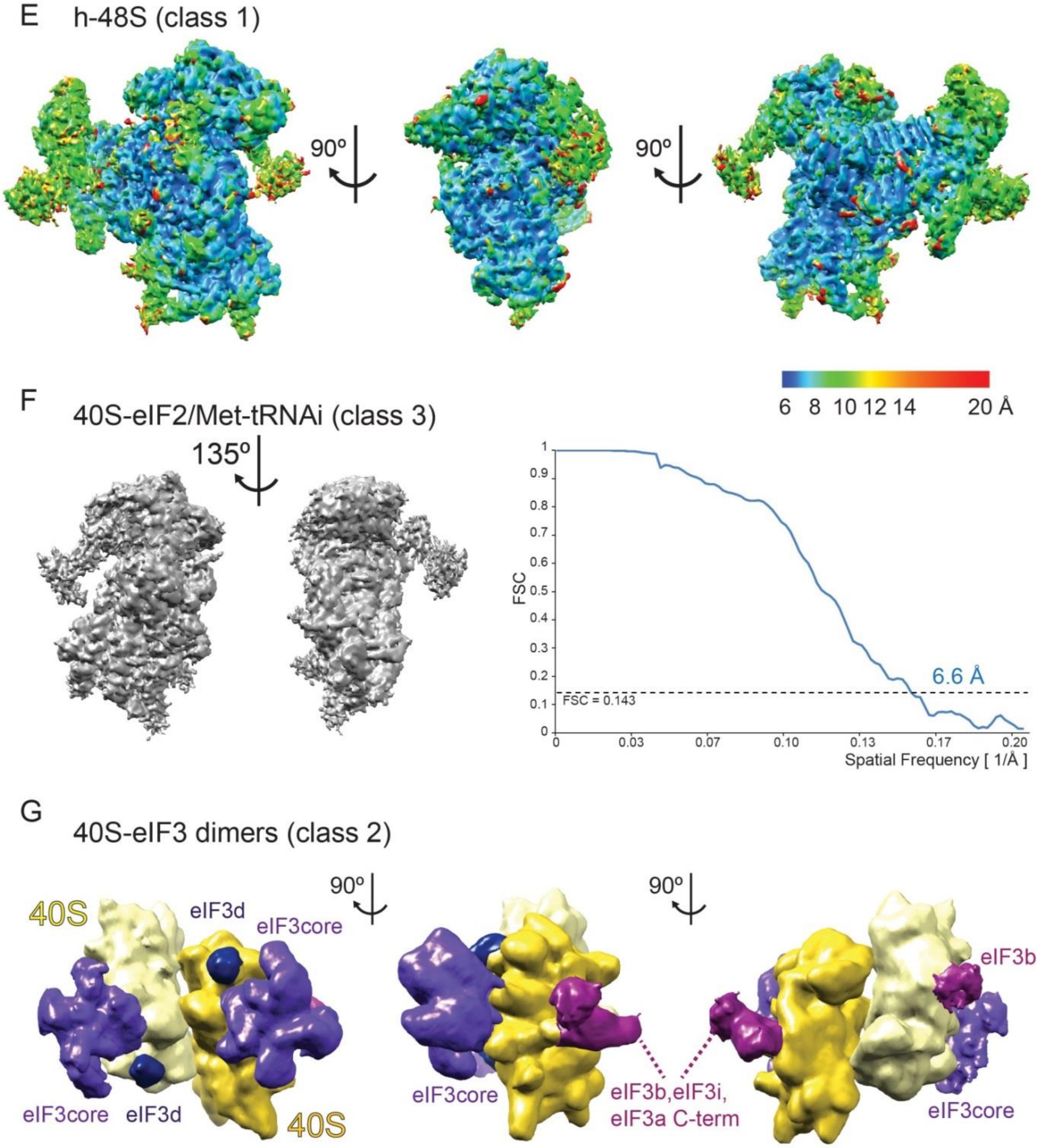

Supplementary Figure S2. h-48S complex cryo-EM data processing. A. Representative micrograph of the human 48S sample. **B.** Selected cryo-EM 2D class averages of h-48S particles. **C.** Maximum likelihood 3D classification scheme. The population of each class is indicated by the number of particles and the percentage of the total number of particles. **D.** Fourier Shell Correlation (FSC) curve after gold-standard refinement for the h-48S (class 1). **E.** Cryo-EM reconstruction of the h-48S coloured according to the local resolution estimated using ResMap (see Methods) from three different views. **F.** Left: cryo-EM structure of the 40S-eIF2/Met-tRNAi complex (class 3) after gold-standard refinement. Right: FSC curve for this volume. **G.** Cryo-EM structure of the 40S-eIF3 dimer (class 2) after refinement. Human 40S is coloured yellow, eIF3 subunits and core in purple.

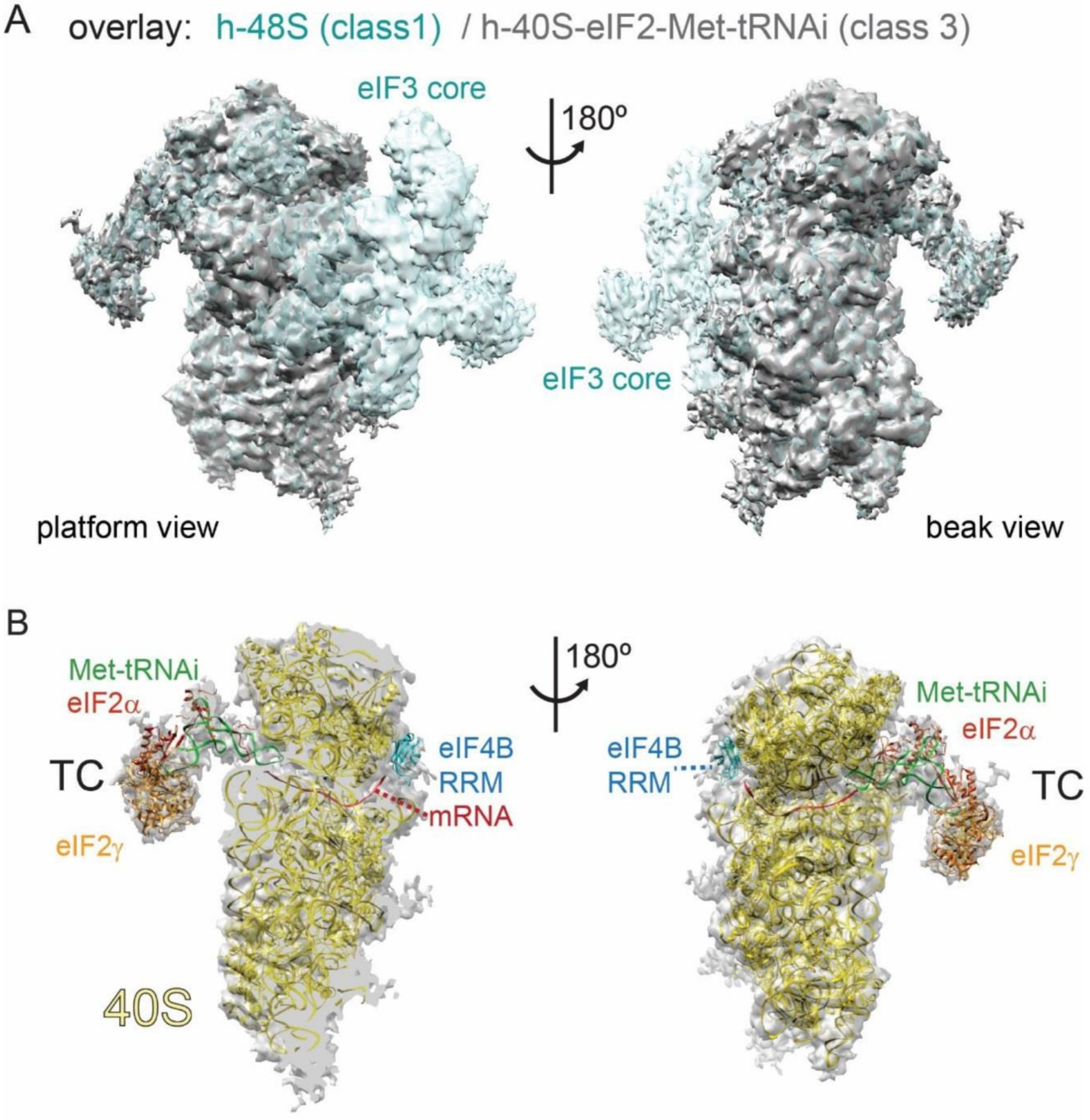

Supplementary Figure S3. Structure of the 40S-eIF2-Met-tRNAi initiation complex. A. Overlay of the h-48S structure (class 1; density shown in transparent cyan) with the 40S-eIF2-Met-tRNAi initiation complex (class 3; density shown in grey). The 40S and the TC are in a virtually identical conformation in both 3D reconstructions. The 40S-eIF2-Met-tRNAi lacks the density for all eIF3 subunits. **B.** Atomic model of the 40S-eIF2-Met-tRNAi initiation complex in a platform view (left) and a beak view (right) highlighting the presence of the TC, mRNA and eIF4B (cyan) in this structure. The 40S subunit is coloured yellow, mRNA red, Met-tRNAi green, eIF2α orange-red and eIF2γ orange.

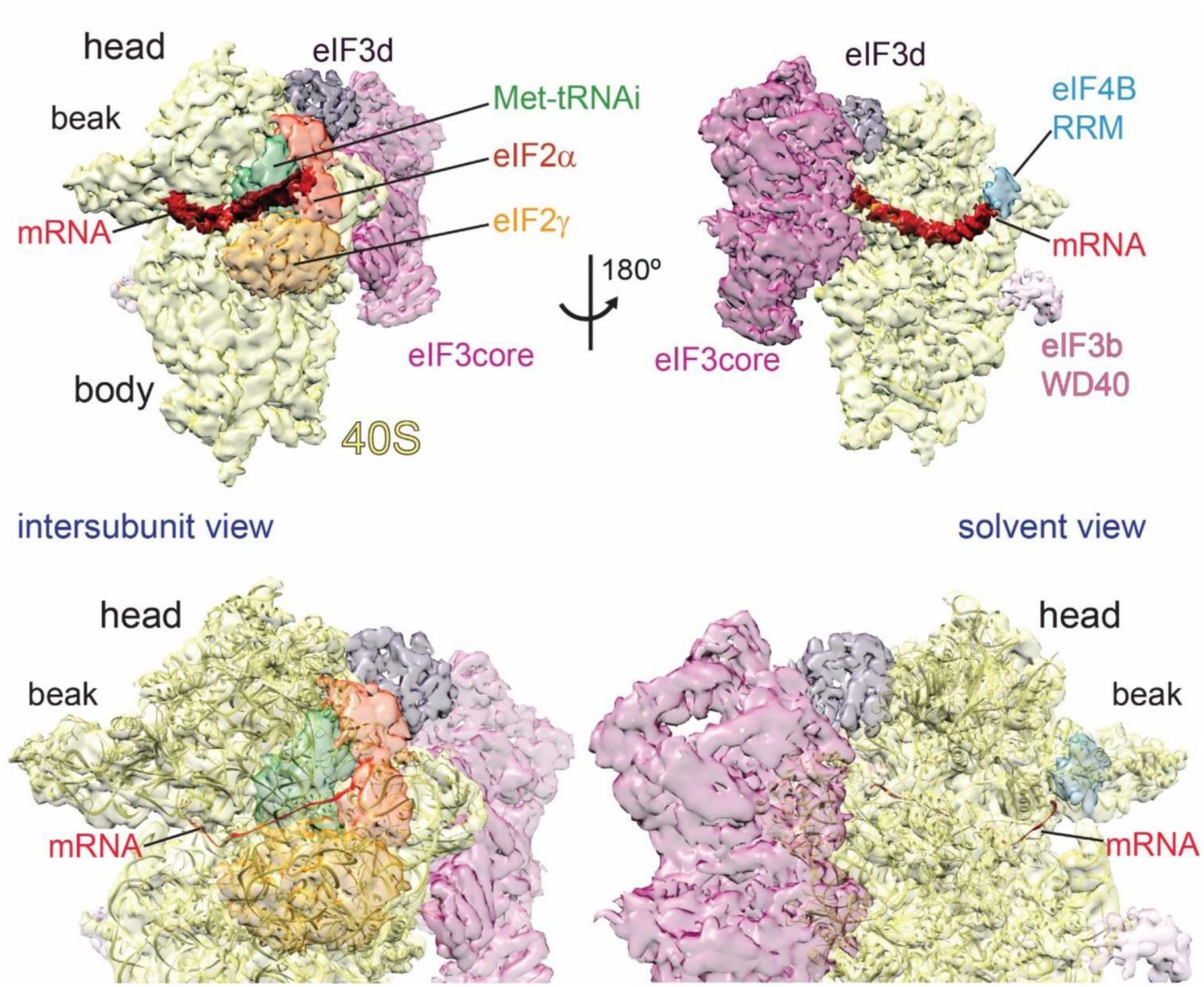

Supplementary Figure S4: Path of the mRNA in the human 48S complex structure. The 40S subunit density is coloured transparent yellow, mRNA solid red, Met-tRNAi transparent green, eIF2α transparent orange-red, eIF2γ transparent orange and eIF3 transparent purple. Below a close-up view is shown with the models of the 40S subunit (yellow) and the mRNA (red) (cartoon representation).

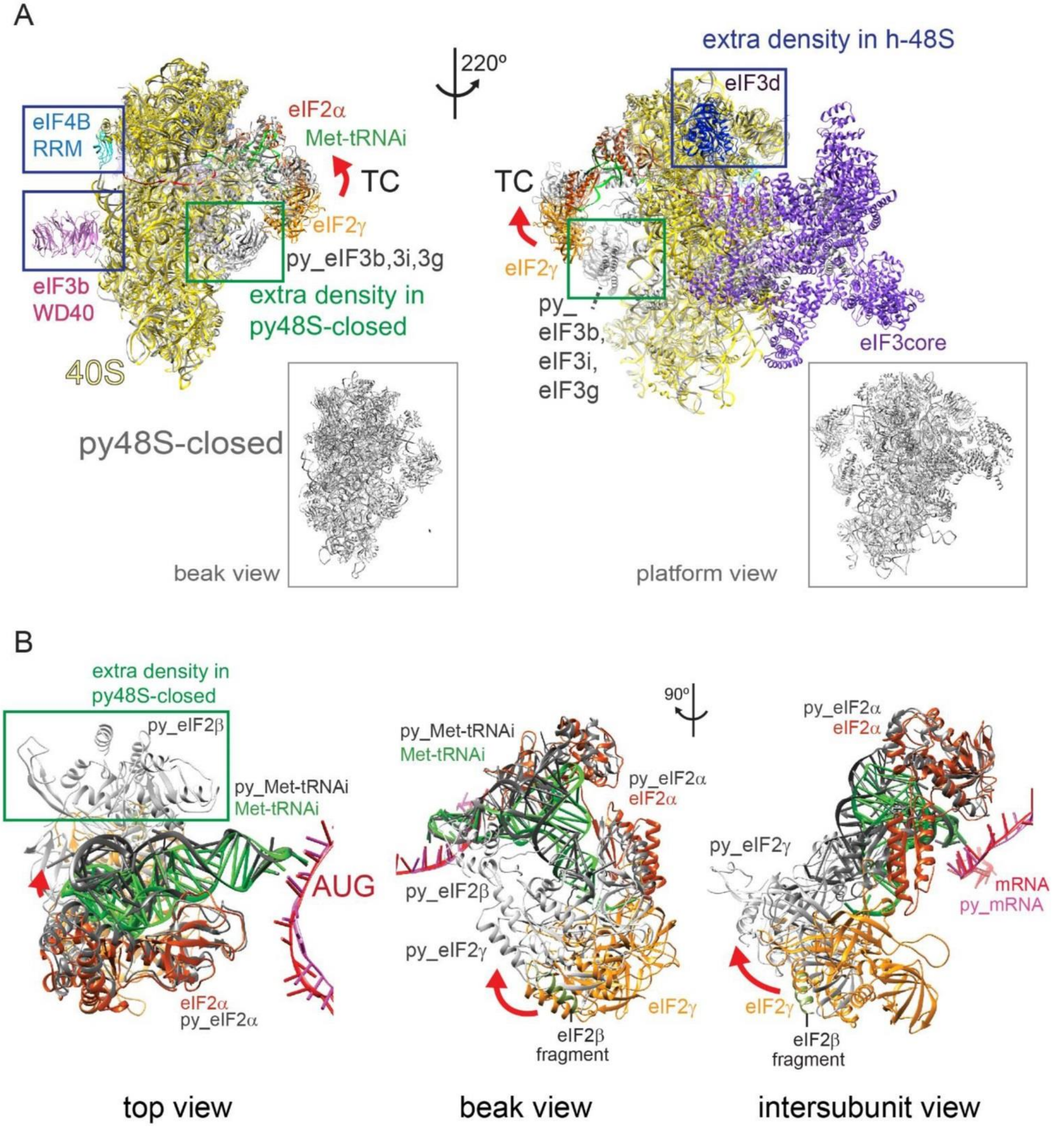
A
220º
extra density in h-48S
eIF3d
eIF4B
RRM
eIF2α
Met-tRNAi
TC
TC
eIF3b
WD40
eIF2γ
py_eIF3b,3i,3g
eIF2γ
extra density in
py48S-closed
py_
eIF3b,
eIF3i,
eIF3g
eIF3core
40S
py48S-closed
beak view
platform view
B
extra density in
py48S-closed
py_eIF2β
py_Met-tRNAi
Met-tRNAi
AUG
eIF2α
py_eIF2α
90º
py_Met-tRNAi
Met-tRNAi
py_eIF2α
eIF2α
py_eIF2β
py_eIF2γ
eIF2γ
eIF2β
fragment
py_eIF2α
eIF2α
py_eIF2γ
mRNA
py_mRNA
eIF2γ
eIF2β
fragment
top view
beak view
intersubunit view

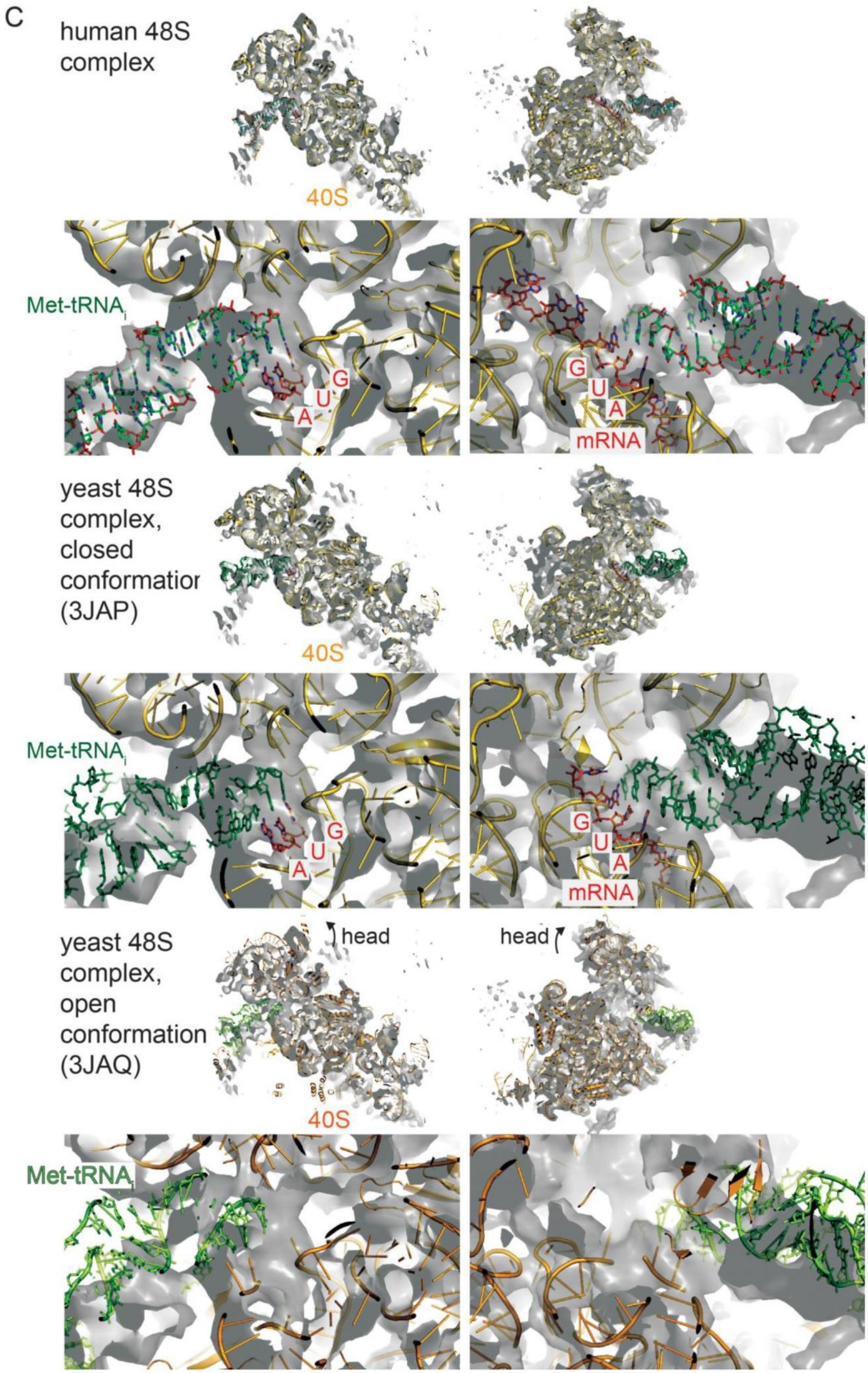
C
human 48S complex
40S
Met-tRNAi
G
U
A
mRNA
yeast 48S complex, closed conformation (3JAP)
40S
Met-tRNAi
G
U
A
mRNA
yeast 48S complex, open conformation (3JAQ)
head
head
40S
Met-tRNAi

Supplementary Figure S5. Comparison of the yeast pre-IC 48S with the human 48S pre-IC structure. A. Overlay of the yeast py48S-closed structure (grey) (4) with the h-48S complex (this study) in a beak view (left) and a platform view (right). Conformational changes in the TC are highlighted by a red arrow. Proteins found only in the py48S-closed structure (4) at a specific position are highlighted by a green box; proteins present only in the h-48S structure at a specific position are highlighted by a blue box. Insets: yeast py48S-closed structure alone in the same view. **B.** Zoom into the conformation of the TC from the top view (left), the beak view (middle) and the intersubunit view (right), highlighting the codon-anticodon pairing and the conformational differences of the TC (Met-tRNAi and eIF2 subunits) in the two 48S structures. In the yeast py48S-closed structure, the eIF2beta subunit is well-defined compared to the h-48S structure. The h-48S subunits are coloured as in Supplementary Figure S3; eIF3 subunits in purple (core), darkblue (eIF3d) and pink (eIF3b). The yeast py48S-closed pre-IC complex is shown in grey, except in panel B where the py_mRNA is coloured pink. **C.** Close-up views of the P-side of the h-48S structure. For orientation, the complete 48S structure is shown in the same view above the close-up view. Top: the atomic model of human 40S, initiator Met-tRNA and mRNA fitted into the h-48S density. The mRNA AUG start codon is labelled. Middle: yeast 40S, initiator Met-tRNA and mRNA model of the yeast py48S complex in the closed conformation (4) fitted into the h-48S density. The mRNA AUG start codon is labelled. Below: yeast 40S and initiator Met-tRNA model of the yeast py48S complex in the open conformation (4) fitted into the h-48S density. When the 40S body of the py-48S open conformation structure (P_{out}) is fitted into the h-48S density, the 40S head domain and the Met-tRNAi are not accommodated in the density. Arrows indicate the conformational change of the head domain in the open py_48S conformation compared to the closed conformation and the h-48S structure.

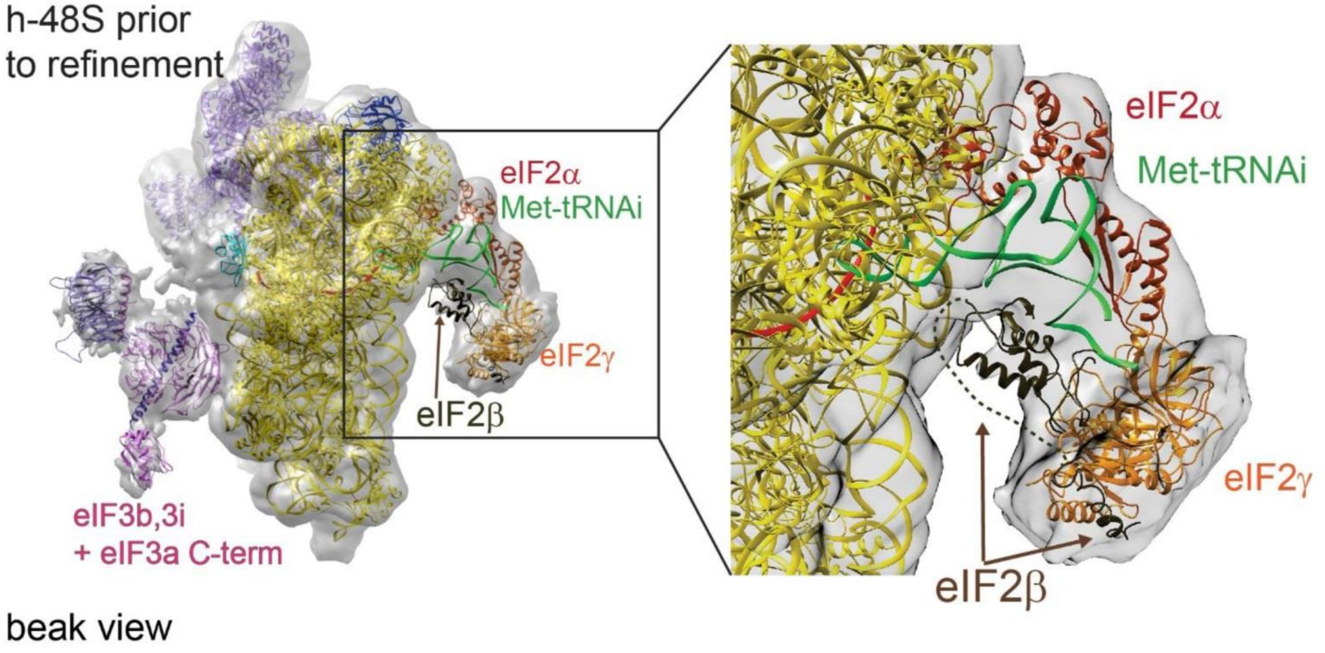

Supplementary Figure S6. Localization of eIF2β in the h-48S low-resolution cryo-EM structure prior to refinement. Density attributed to eIF2 β is located next to Met-tRNAi, between eIF2γ and the 40S subunit and bound to eIF2 γ. The h-48S subunits are coloured as described in Supplementary Figure S3. The YLC subunits of eIF3 are coloured pink (eIF3b), purple (eIF3i) and dark blue (C-terminal part of eIF3a). Flexible parts of eIF2β (in olive colour) are indicated by a dotted line. Left: beak view. Right: zoom into the TC.

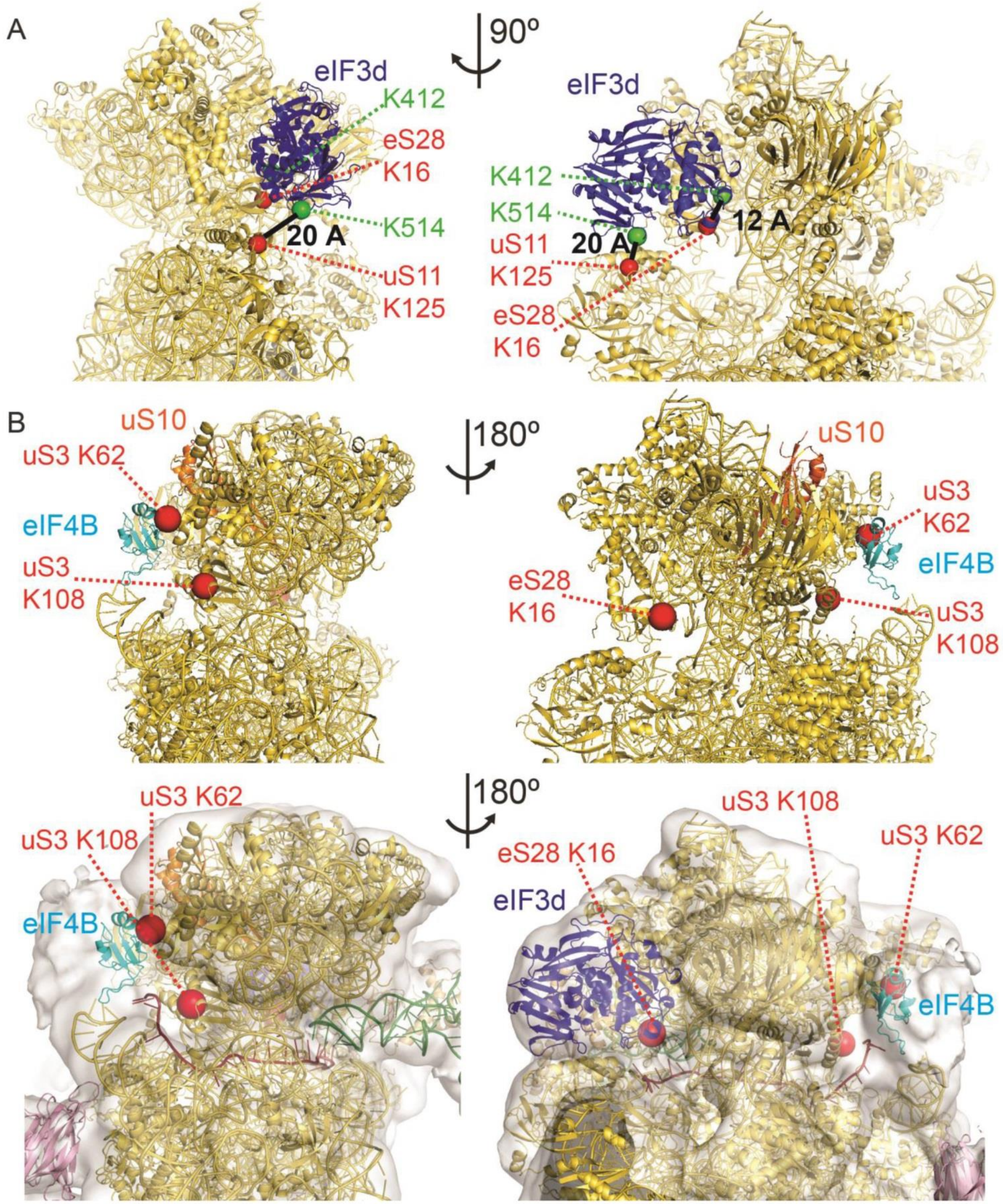

Supplementary Figure S7. Cross-linking-Mass Spectrometry results support the localization of eIF3d and eIF4B in the h-48S complex. A. Visualization of the 40S-eIF3d cross-linking results. The cross-linked lysines in the 40S subunit are depicted as red spheres and in eIF3d as green spheres. The

distance between the cross-linked residues in the atomic model is indicated. Residue 149 of eIF3d is not part of the atomic model. **B.** Cross-linking sites of eIF4B on the 40S subunit are shown as red spheres. Residues 62 and 108 of ribosomal protein uS3 are close to the density attributed to the eIF4B RRM (atomic model in cyan). Structural information for the cross-linked residues of eIF4B is not available. The cross-links of eIF4B with uS3 and eS28 (both well above the xQuest score cut-off for false discovery) could indicate that eIF4B stretches along the 40S subunit and binds next to the mRNA channel entry with its N-terminal RRM and near the mRNA channel exit with its basic domain. Below: atomic model and h-48S EM density prior to refinement. Additional density is detected next to the RRM domain of eIF4A, but not next to ribosomal protein eS28. EM density is shown in transparent grey.

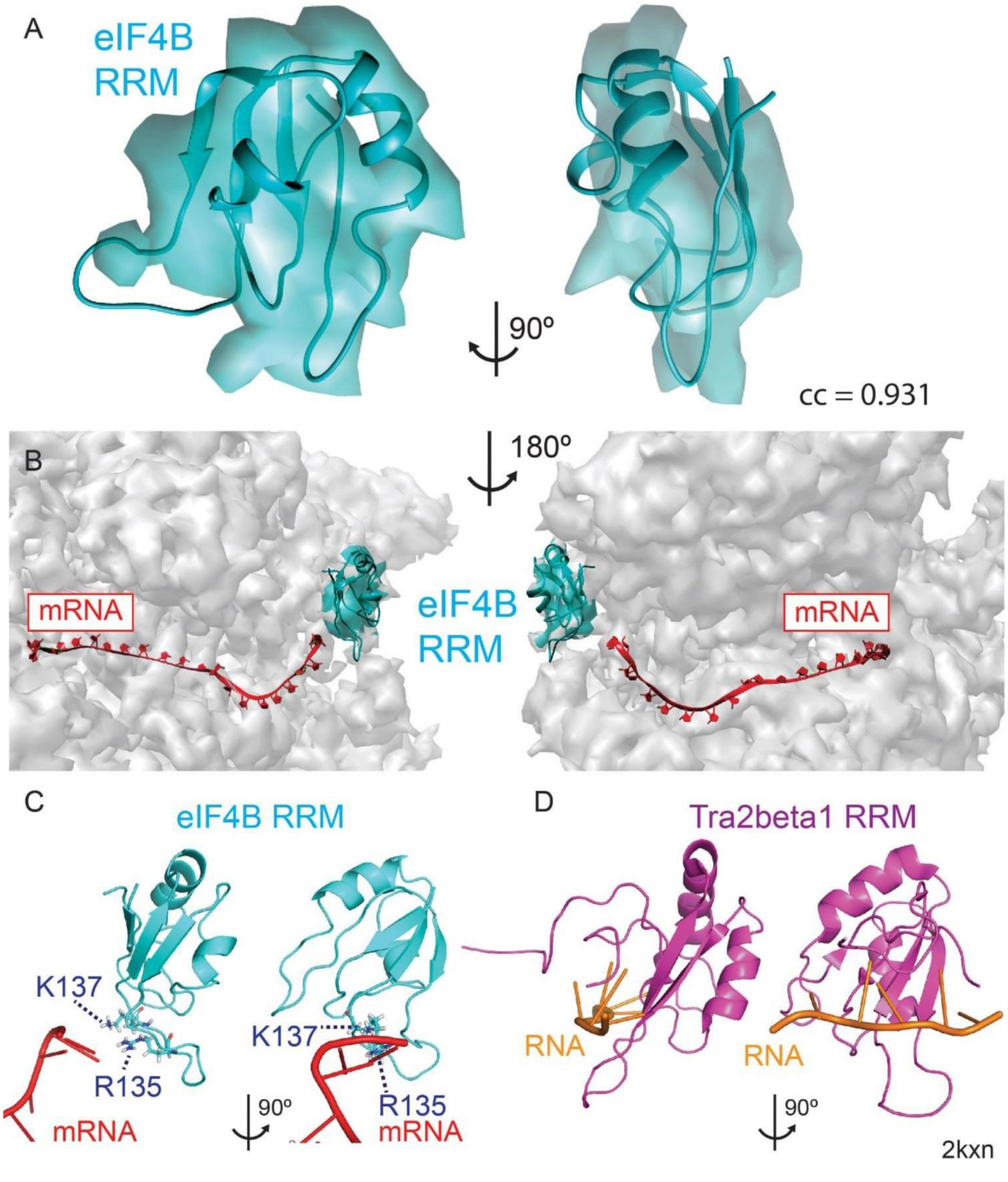

Supplementary Figure S8. Fitting of the eIF4B RRM domain. A. Fitting of the NMR structure of the eIF4B RRM domain (PDB ID 2J76) (36) into the corresponding EM density. The best fit in Chimera yields a cross correlation value of 0.931. For comparison, the atomic model of the eIF3 PCI/MPN core (filtered to 10 Å which is the average local resolution of this part) and the corresponding density of the

map have a cross-correlation value of 0.939. **B.** Positions of the eIF4B RRM (cyan) and the mRNA (red) in the h-48S structure (density in transparent grey). **C.** Close-up view of the eIF4B RRM atomic model and the mRNA model. The conserved residues required for single-stranded RNA binding (Ref. 10 and references therein) are shown as sticks. **D.** To assess the RNA interaction mode of the eIF4B RRM, an unrelated RRM-RNA complex (human Tra2beta1 RRM in complex with AAGAAC RNA) is shown, indicating a similar position of the RNA-binding site (64).

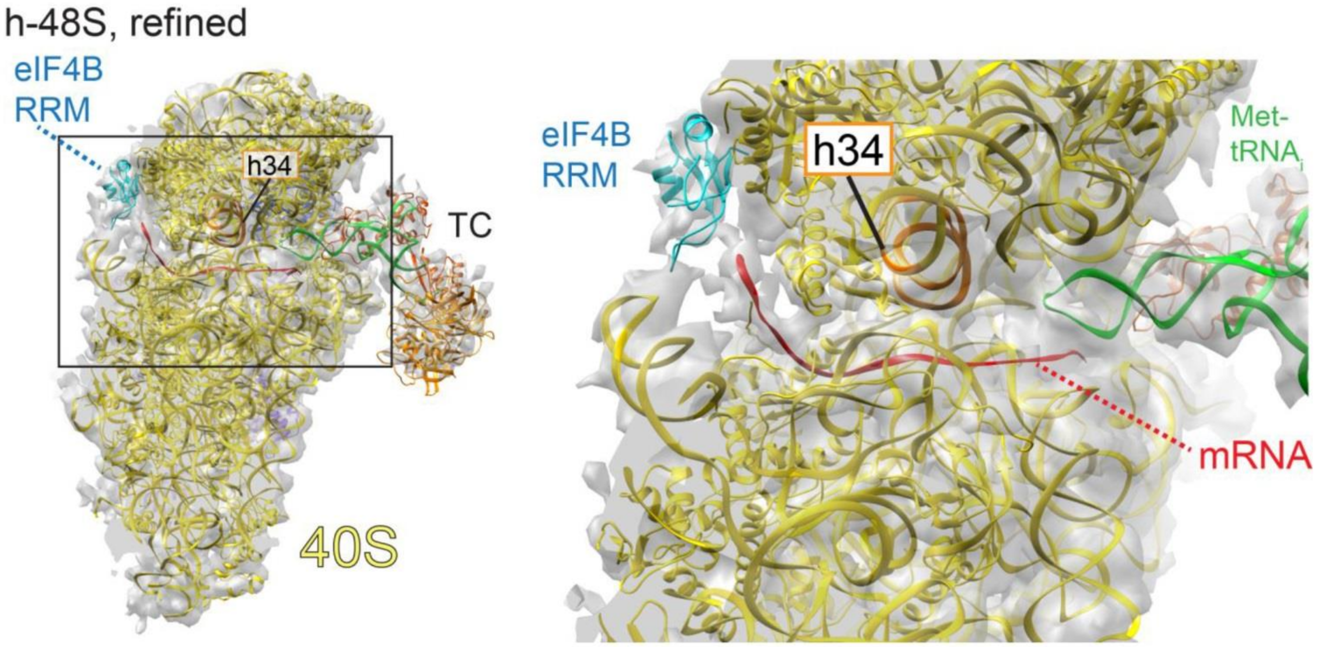

Supplementary Figure S9. eIF4B binding to 40S does not induce conformational changes in ribosomal RNA helix 34 (highlighted in orange), indicating that eIF4B stabilizes mRNA binding to the complex by contacting directly the mRNA and the 40S subunit. Left: beak view, right: zoom into the area highlighted by the grey box in the left panel. Subunits are coloured as described in Supplementary Figure S3.

Supplementary References

62. Namy, O., Moran, S.J., Stuart, D.I., Gilbert, R.J. and Brierley, I. (2006) A mechanical explanation of RNA pseudoknot function in programmed ribosomal frameshifting. *Nature*, **441**, 244-247.

63. Shirokikh, N.E., Alkalaeva, E.Z., Vassilenko, K.S., Afonina, Z.A., Alekhina, O.M., Kisselev, L.L. and Spirin, A.S. (2010) Quantitative analysis of ribosome-mRNA complexes at different translation stages. *Nucleic Acids Res.*, **38**, e15.

64. Clery, A., Jayne, S., Benderska, N., Dominguez, C., Stamm, S. and Allain, F.H. (2011) Molecular basis of purine-rich RNA recognition by the human SR-like protein Tra2-beta1. *Nat. Struct. Mol. Biol.*, **18**, 443-450.
