# Supplementary material for: Structure of a human cap-dependent 48S translation pre-initiation complex
Source: Nucleic Acids Res. 2018 Feb 1;46(5):2678–89. doi: 10.1093/nar/gky054 (PMC5861459; doi:10.1093/nar/gky054)
Supplement: Supplementary Data [file gky054_supp.zip › nar-01614-r-2017-File008.pdf]

**Description of the column headers**

| | |
|---|---|
| **Id** | Assigned peptides and cross-linking sites within the peptide sequences. The longer peptide is designated as (a)lpha, the shorter as (b)eta. |
| **Protein** | SwissProt/UniProt accession number and identifier of the protein |
| **AbsPos1** | Position in the protein sequence of protein 1 |
| **AbsPos2** | Position in the protein sequence of protein 2 |
| **Mr** | Molecular mass calculated from experimental m/z and z (neutral mass) |
| **Mz** | Experimentally observed mass-to-charge ratio of the precursor ion in Da |
| **z** | Experimentally observed precursor charge |
| **Error_rel[ppm]** | Deviation between experimental and theoretical mass in ppm |
| **Id-score** | Identification score as assigned by xQuest |
