# Supplementary material for: Structure of a human cap-dependent 48S translation pre-initiation complex
Source: Nucleic Acids Res. 2018 Feb 1;46(5):2678–89. doi: 10.1093/nar/gky054 (PMC5861459; doi:10.1093/nar/gky054)
Supplement: Supplementary Data [file gky054_supp.zip › nar-01614-r-2017-File009.pdf]

	A	B	C	D	E	F	G	H	I	J	K
1	(A) Supplementary Table S1, listing the inter-molecular Lys-Lys cross-links by DSS in the human 48S complex identified in XL-MS.										
2	Id	Protein1	Protein2	AbsPos1	AbsPos2	Mr	Mz	z	Error_rel[ppm]	Id-Score	Comments
3	**TKTPGPGAQSALR-KFVIR-a2-b1**	**sp|P62263|RS14_HUMAN**	**sp|P62854|RS26_HUMAN**	**106**	**38**	**2082.19**	**695.071**	**3**	**-2.5**	**39.49**	**high/high data set**
4	**IGKFEAR-LLQKNNK-a3-b4**	**sp|Q13347|EIF3I_HUMAN**	**sp|P55884|EIF3B_HUMAN**	**264**	**693**	**1814.032**	**454.516**	**4**	**-5**	**39.32**	
5	**CSKEVATAIR-AKSMK-a3-b2**	**sp|P15880|RS2_HUMAN**	**sp|P23396|RS3_HUMAN**	**145**	**148**	**1834.958**	**612.661**	**3**	**-3.5**	**39.17**	
6	**LKYALTGDEVKK-KFMTNR-a2-b1**	**sp|P62701|RS4X_HUMAN**	**sp|P62847|RS24_HUMAN**	**53**	**11**	**2297.244**	**575.319**	**4**	**-0.6**	**38.68**	
7	**YKLLGGLAVR-KGPNANS-a2-b1**	**sp|P23396|RS3_HUMAN**	**sp|P62861|RS30_HUMAN**	**108**	**53**	**1913.065**	**638.696**	**3**	**-4.6**	**38.48**	
8	**EKGYIDLSK-VAKSNR-a2-b3**	**sp|P05198|IF2A_HUMAN**	**sp|P46782|RS5_HUMAN**	**80**	**201**	**1863**	**622.008**	**3**	**-5.3**	**38.17**	
9	**DVKDGK-KFTYR-a3-b1**	**sp|P62269|RS18_HUMAN**	**sp|P62841|RS15_HUMAN**	**91**	**14**	**1511.796**	**378.957**	**4**	**-1.6**	**38.08**	
10	**EKQVPVDVVEMK-KQVVNIPSFIVR-a2-b1**	**sp|P55884|EIF3B_HUMAN**	**sp|P46781|RS9_HUMAN**	**552**	**139**	**2936.625**	**735.164**	**4**	**-5.4**	**38.03**	
11	**LKYALTGDEVK-KFMTNR-a2-b1**	**sp|P62701|RS4X_HUMAN**	**sp|P62847|RS24_HUMAN**	**53**	**11**	**2169.142**	**724.055**	**3**	**-3.7**	**37.78**	
12	**EKNPDMVAGEK-IDLVKESQAK-a2-b5**	**sp|P20042|IF2B_HUMAN**	**sp|P41091|IF2G_HUMAN**	**190**	**196**	**2484.261**	**829.095**	**3**	**-7**	**37.73**	
13	**TKSLSFR-TKSMFQR-a2-b2**	**sp|P60228|EIF3E_HUMAN**	**sp|Q9Y262|EIF3L_HUMAN**	**409**	**347**	**1871.988**	**469.005**	**4**	**-2.7**	**37.46**	
14	**SRLDQELKLIGEYGLR-KGPNANS-a8-b1**	**sp|P46781|RS9_HUMAN**	**sp|P62861|RS30_HUMAN**	**30**	**53**	**2713.421**	**905.481**	**3**	**-7.1**	**37.29**	
15	**EKQVPVDVVEMK-LAAKQSSG-a2-b4**	**sp|P55884|EIF3B_HUMAN**	**sp|P62701|RS4X_HUMAN**	**552**	**259**	**2298.21**	**767.078**	**3**	**-1.7**	**37.24**	
16	**QKLDSQR-VTKVLGR-a2-b3**	**sp|O15371|EIF3D_HUMAN**	**sp|P62857|RS28_HUMAN**	**412**	**16**	**1783.026**	**595.35**	**3**	**-3.1**	**37.18**	
17	**AASIFGGAKPVDTAAR-VTKVLGR-a9-b3**	**sp|P23588|IF4B_HUMAN,db|PROT04|IF4B_HUM**	**sp|P62857|RS28_HUMAN**	**394**	**16**	**2440.375**	**611.102**	**4**	**-2**	**37.16**	
18	**DSDKTDTDWR-TQNVLGEKGR-a4-b8**	**sp|P23588|IF4B_HUMAN,db|PROT04|IF4B_HUM**	**sp|P23396|RS3_HUMAN**	**223**	**62**	**2476.185**	**826.403**	**3**	**0.9**	**37.13**	
19	**FVTGNKILR-SFQKIQVR-a6-b4**	**sp|P62277|RS13_HUMAN**	**sp|P62081|RS7_HUMAN**	**70**	**74**	**2189.262**	**730.762**	**3**	**-2.8**	**36.98**	
20	**GYVKEQFAWR-TQNVLGEKGR-a4-b8**	**sp|P46783|RS10_HUMAN**	**sp|P23396|RS3_HUMAN**	**59**	**62**	**2521.299**	**841.441**	**3**	**-3.4**	**36.97**	
21	**TKSLSFR-EKYGDK-a2-b2**	**sp|P60228|EIF3E_HUMAN**	**sp|Q9Y262|EIF3L_HUMAN**	**409**	**393**	**1713.887**	**429.48**	**4**	**-3.7**	**36.92**	
22	**KSDGIYIINLKR-ADGIVSKNF-a11-b7**	**sp|P08865|RSSA_HUMAN**	**sp|P63220|RS21_HUMAN**	**52**	**81**	**2506.37**	**627.6**	**4**	**-4**	**36.88**	
23	**KPLPDHVSIVEPK-NVKSLEK-a1-b3**	**sp|P23396|RS3_HUMAN**	**sp|P60866|RS20_HUMAN**	**202**	**30**	**2412.351**	**483.478**	**5**	**-4.8**	**36.86**	
24	**GQKIVSCR-LLQKNNK-a3-b4**	**sp|O75821|EIF3G_HUMAN**	**sp|P55884|EIF3B_HUMAN**	**153**	**693**	**1941.079**	**648.034**	**3**	**-2.1**	**36.86**	
25	**DPNKQVIR-SGMKIGR-a4-b4**	**sp|O15371|EIF3D_HUMAN**	**sp|P62263|RS14_HUMAN**	**514**	**125**	**1854.006**	**619.01**	**3**	**-4.4**	**36.81**	
26	**FQQKQIENVLR-LEAMCFDGVKR-a4-b10**	**sp|P20042|IF2B_HUMAN**	**sp|P47813|IF1AX_HUMAN,db|PROT02|IF1A**	**265**	**90**	**2864.454**	**717.121**	**4**	**-4.8**	**36.72**	
27	**EKEELEQR-LMEVCKEK-a2-b6**	**sp|Q14152|EIF3A_HUMAN**	**sp|O15372|EIF3H_HUMAN**	**591**	**178**	**2233.085**	**559.279**	**4**	**-5.4**	**36.72**	
28	**SAVPPGADKK-TQNVLGEKGR-a9-b8**	**sp|P46783|RS10_HUMAN**	**sp|P23396|RS3_HUMAN**	**138**	**62**	**2207.183**	**736.736**	**3**	**-3.5**	**36.72**	
29	**FQQKQIENVLR-IDLVKESQAK-a4-b5**	**sp|P20042|IF2B_HUMAN**	**sp|P41091|IF2G_HUMAN**	**265**	**196**	**2669.463**	**668.374**	**4**	**-4.5**	**36.7**	
30	**LLEPVLLLGKER-IAGQVAAANKK-a10-b10**	**sp|P62249|RS16_HUMAN**	**sp|P39019|RS19_HUMAN**	**60**	**143**	**2586.541**	**863.188**	**3**	**-2.5**	**36.7**	
31	**TQGTKIASDGLK-VTKVLGR-a5-b3**	**sp|P61247|RS3A_HUMAN**	**sp|P62857|RS28_HUMAN**	**56**	**16**	**2127.221**	**532.813**	**4**	**-2.3**	**36.61**	
32	**AKELLGQGLLLR-KLCDNLR-a2-b1**	**sp|Q99613|EIF3C_HUMAN**	**sp|Q14152|EIF3A_HUMAN**	**643**	**196**	**2365.348**	**592.345**	**4**	**-1.6**	**36.6**	
33	**ASTSKSESSQK-GNKPWISLPR-a5-b3**	**sp|P62753|RS6_HUMAN**	**sp|P62701|RS4X_HUMAN**	**243**	**233**	**2443.261**	**611.823**	**4**	**-3.9**	**36.53**	
34	**QYAKDIGFIK-NVKSLEK-a4-b3**	**sp|P62273|RS29_HUMAN**	**sp|P60866|RS20_HUMAN**	**48**	**30**	**2136.169**	**535.05**	**4**	**-6.6**	**36.33**	
35	**IDLVKESQAK-NPDMVAGEKR-a5-b9**	**sp|P41091|IF2G_HUMAN**	**sp|P20042|IF2B_HUMAN**	**196**	**199**	**2383.232**	**596.816**	**4**	**-4.2**	**36.27**	
36	**IGKPHTVPCK-IGVLDEGKMK-a3-b8**	**sp|P15880|RS2_HUMAN**	**sp|P46781|RS9_HUMAN**	**176**	**91**	**2362.266**	**591.574**	**4**	**-3.9**	**36.19**	
37	**VKLAVLK-VDDNKK-a2-b5**	**sp|P62979|RS27A_HUMAN**	**sp|P25398|RS12_HUMAN**	**99**	**83**	**1624.97**	**407.25**	**4**	**-4.2**	**36.15**	
38	**EGVMVAKK-KFGQGSR-a7-b1**	**sp|P46783|RS10_HUMAN**	**sp|P62273|RS29_HUMAN**	**24**	**13**	**1776.95**	**593.325**	**3**	**-3**	**36.15**	
39	**SDGIYIINLKR-LAKADGIVSK-a10-b3**	**sp|P08865|RSSA_HUMAN**	**sp|P63220|RS21_HUMAN**	**52**	**74**	**2429.383**	**608.354**	**4**	**-2.5**	**36.03**	
40	**KAEAGAGSATEFQFR-TQNVLGEKGR-a1-b8**	**sp|P46783|RS10_HUMAN**	**sp|P23396|RS3_HUMAN**	**139**	**62**	**2807.407**	**702.86**	**4**	**-4.7**	**35.96**	
41	**KGQGGAGAGDDEEED-SLKISGIK-a1-b3**	**sp|P46781|RS9_HUMAN**	**sp|P55884|EIF3B_HUMAN**	**180**	**463**	**2416.147**	**806.39**	**3**	**-5.5**	**35.68**	
42	**FVNVVPTFGKK-YKLLGGLAVR-a10-b2**	**sp|P62861|RS30_HUMAN**	**sp|P23396|RS3_HUMAN**	**51**	**108**	**2461.448**	**616.37**	**4**	**0.8**	**35.58**	
43	**NTKGGDAPAAGEDA-IAGQVAAANKK-a3-b10**	**sp|P62851|RS25_HUMAN**	**sp|P39019|RS19_HUMAN**	**114**	**143**	**2480.239**	**827.754**	**3**	**-4.7**	**35.54**	
44	**MKLDYILGLK-LAAKQSSG-a2-b4**	**sp|P46781|RS9_HUMAN**	**sp|P62701|RS4X_HUMAN**	**93**	**259**	**2091.158**	**698.061**	**3**	**-3.2**	**35.49**	
45	**SDGIYIINLKR-ADGIVSKNF-a10-b7**	**sp|P08865|RSSA_HUMAN**	**sp|P63220|RS21_HUMAN**	**52**	**81**	**2378.277**	**793.767**	**3**	**-3.2**	**35.44**	
46	**NNGKIELIK-LAAKQSSG-a4-b4**	**sp|P55884|EIF3B_HUMAN**	**sp|P62701|RS4X_HUMAN**	**599**	**259**	**1926.077**	**643.034**	**3**	**-0.7**	**35.39**	
47	**VTKVLGR-QKWDQK-a3-b2**	**sp|P62857|RS28_HUMAN**	**sp|O15371|EIF3D_HUMAN**	**16**	**149**	**1740.98**	**581.334**	**3**	**-5.2**	**35.2**	
48	**EKNPDMVAGEKR-IDLVKESQAK-a11-b5**	**sp|P20042|IF2B_HUMAN**	**sp|P41091|IF2G_HUMAN**	**199**	**196**	**2640.37**	**661.1**	**4**	**-3.6**	**35.16**	
49	**QFQKF-YYKTK-a4-b3**	**sp|P62280|RS11_HUMAN**	**sp|P62277|RS13_HUMAN**	**157**	**130**	**1535.797**	**512.94**	**3**	**-3.5**	**34.99**	
50	**FRPAGAAPRPPPKPM-VTKVLGR-a13-b3**	**sp|P62854|RS26_HUMAN**	**sp|P62857|RS28_HUMAN**	**113**	**16**	**2498.421**	**625.613**	**4**	**-4.1**	**34.95**	
51	**SVPTWLKLTSDDVK-SFQKIQVR-a7-b4**	**sp|P62277|RS13_HUMAN**	**sp|P62081|RS7_HUMAN**	**27**	**74**	**2730.485**	**683.629**	**4**	**-3.9**	**34.85**	
52	**DGKYSQVLANGLDNK-ISGGNDKQGFPMK-a3-b7**	**sp|P62269|RS18_HUMAN**	**sp|P62753|RS6_HUMAN**	**94**	**58**	**3136.535**	**785.141**	**4**	**-4.9**	**34.83**	
53	**LGEWVGLCKIDR-MLMPKK-a9-b5**	**sp|P25398|RS12_HUMAN**	**sp|P46783|RS10_HUMAN**	**93**	**5**	**2329.233**	**583.316**	**4**	**-1.5**	**34.81**	
54	**QLDEPKLER-VTKVLGR-a6-b3**	**sp|P23588|IF4B_HUMAN,db|PROT04|IF4B_HUM**	**sp|P62857|RS28_HUMAN**	**424**	**16**	**2036.156**	**510.047**	**4**	**-3.6**	**34.63**	
55	**VADWTGATYQDKR-NTSKQQQQK-a12-b4**	**sp|O15371|EIF3D_HUMAN**	**sp|O15372|EIF3H_HUMAN**	**53**	**269**	**2736.334**	**685.091**	**4**	**-4.6**	**34.51**	
56	**GIYAYGFEKPSAIQQR-LKEELEEAR-a9-b2**	**sp|P60842.1|IF4A1_HUMAN,db|PROT03|IF4A1_**	**sp|Q04637|IF4G1_HUMAN,db|PROT05|IF4(**	**54,72**	**914**	**3080.572**	**771.151**	**4**	**-3.4**	**34.35**	
57	**EEQAAAEKAVTK-VTKVLGR-a8-b3**	**sp|P08865|RSSA_HUMAN**	**sp|P62857|RS28_HUMAN**	**220**	**16**	**2183.21**	**546.81**	**4**	**-2.6**	**34.3**	
58	**ELLTLDEKDPR-FDVQLKDLEK-a8-b6**	**sp|P46781|RS9_HUMAN**	**sp|P62244|RS15A_HUMAN**	**66**	**84**	**2699.419**	**675.863**	**4**	**-3**	**34.13**	
59	**EKDFSPEALK-EKEELEQR-a2-b2**	**sp|O15372|EIF3H_HUMAN**	**sp|Q14152|EIF3A_HUMAN**	**180**	**591**	**2360.164**	**591.049**	**4**	**-4.4**	**33.5**	
60	**MCSMVKK-KFVIR-a6-b1**	**sp|P61247|RS3A_HUMAN**	**sp|P62854|RS26_HUMAN**	**115**	**38**	**1681.901**	**421.483**	**4**	**-4.5**	**32.94**	
61	**LDSQKHIDFSLR-LAAKQSSG-a5-b4**	**sp|P46781|RS9_HUMAN**	**sp|P62701|RS4X_HUMAN**	**155**	**259**	**2356.234**	**590.066**	**4**	**-1.9**	**32.83**	
62	**QMVIDVLHPGKATVPK-KGQGGAGAGDDEEED-a11-b1**	**sp|P62847|RS24_HUMAN**	**sp|P46781|RS9_HUMAN**	**32**	**180**	**3303.585**	**826.904**	**4**	**-2.3**	**30.42**	
63											
64	**DPNKQVIR-SGMKIGR-a4-b4**	**sp|O15371|EIF3D_HUMAN**	**sp|P62263|RS14_HUMAN**	**514**	**125**	**1854.009**	**464.51**	**4**	**-2.9**	**33.69**	**high/low data set**
65	**TQGTKIASDGLK-VTKVLGR-a5-b3**	**sp|P61247|RS3A_HUMAN**	**sp|P62857|RS28_HUMAN**	**56**	**16**	**2127.216**	**710.08**	**3**	**-4.8**	**32.68**	
66	**IGKFEAR-LLQKNNK-a3-b4**	**sp|Q13347|EIF3I_HUMAN**	**sp|P55884|EIF3B_HUMAN**	**264**	**693**	**1814.035**	**605.686**	**3**	**-3.4**	**31.16**	
67	**FNVLKVTK-KGKDK-a5-b1**	**sp|P62280|RS11_HUMAN**	**db|P55010|IF5_HUMAN_His_tagged**	**144**	**184**	**1659.986**	**416.004**	**4**	**-4.1**	**31.15**	
68	**VKLAVLK-VDDNKK-a2-b5**	**sp|P62979|RS27A_HUMAN**	**sp|P25398|RS12_HUMAN**	**99**	**83**	**1624.969**	**407.25**	**4**	**-4.4**	**30.93**	
69	**KAEAGAGSATEFQFR-TQNVLGEKGR-a1-b8**	**sp|P46783|RS10_HUMAN**	**sp|P23396|RS3_HUMAN**	**139**	**62**	**2807.412**	**702.861**	**4**	**-3**	**30.7**	
70	**FNVLKVTK-KTGGK-a5-b1**	**sp|P62280|RS11_HUMAN**	**sp|P62241|RS8_HUMAN**	**144**	**13**	**1574.931**	**394.741**	**4**	**-5.1**	**30.67**	
71	**VTKVLGR-QKWDQK-a3-b2**	**sp|P62857|RS28_HUMAN**	**sp|O15371|EIF3D_HUMAN**	**16**	**149**	**1740.982**	**436.253**	**4**	**-4**	**30.45**	
72	**DSDKTDTDWR-TQNVLGEKGR-a4-b8**	**sp|P23588|IF4B_HUMAN,db|PROT04|IF4B_HUM**	**sp|P23396|RS3_HUMAN**	**223**	**62**	**2476.172**	**620.051**	**4**	**-4.5**	**30.42**	
73	**FVTGNKILR-SFQKIQVR-a6-b4**	**sp|P62277|RS13_HUMAN**	**sp|P62081|RS7_HUMAN**	**70**	**74**	**2189.261**	**730.761**	**3**	**-3.6**	**29.9**	
74	**GQKIVSCR-LLQKNNK-a3-b4**	**sp|O75821|EIF3G_HUMAN**	**sp|P55884|EIF3B_HUMAN**	**153**	**693**	**1941.076**	**648.033**	**3**	**-3.6**	**29.63**	
75	**TKSLSFR-TKSMFQR-a2-b2**	**sp|P60228|EIF3E_HUMAN**	**sp|Q9Y262|EIF3L_HUMAN**	**409**	**347**	**1871.984**	**469.004**	**4**	**-4.8**	**29.28**	
76	**QKLDSQR-VTKVLGR-a2-b3**	**sp|O15371|EIF3D_HUMAN**	**sp|P62857|RS28_HUMAN**	**412**	**16**	**1783.024**	**446.764**	**4**	**-4.1**	**29.09**	
77	**GYVKEQFAWR-TQNVLGEKGR-a4-b8**	**sp|P46783|RS10_HUMAN**	**sp|P23396|RS3_HUMAN**	**59**	**62**	**2521.3**	**631.333**	**4**	**-2.9**	**28.9**	
78	**VANVSLLALYKGK-GQTPKVAK-a11-b5**	**sp|P62266|RS23_HUMAN**	**sp|P62861|RS30_HUMAN**	**135**	**18**	**2340.369**	**781.131**	**3**	**-3.9**	**28.51**	
79	**EKQVPVDVVEMK-KQVVNIPSFIVR-a2-b1**	**sp|P55884|EIF3B_HUMAN**	**sp|P46781|RS9_HUMAN**	**552**	**139**	**2936.631**	**735.166**	**4**	**-3.2**	**27.78**	
80	**QFQKF-YYKTK-a4-b3**	**sp|P62280|RS11_HUMAN**	**sp|P62277|RS13_HUMAN**	**157**	**130**	**1535.798**	**512.94**	**3**	**-3**	**27.76**	
81	**LLEPVLLLGKER-IAGQVAAANKK-a10-b10**	**sp|P62249|RS16_HUMAN**	**sp|P39019|RS19_HUMAN**	**60**	**143**	**2586.535**	**647.641**	**4**	**-4.9**	**27.49**	
82	**KPLPDHVSIVEPK-NVKSLEK-a1-b3**	**sp|P23396|RS3_HUMAN**	**sp|P60866|RS20_HUMAN**	**202**	**30**	**2412.353**	**604.096**	**4**	**-4**	**27.36**	
83	**FVNVVPTFGKK-YKLLGGLAVR-a10-b2**	**sp|P62861|RS30_HUMAN**	**sp|P23396|RS3_HUMAN**	**51**	**108**	**2461.434**	**616.366**	**4**	**-4.7**	**27.3**	
84	**MKLDYILGLK-LAAKQSSG-a2-b4**	**sp|P46781|RS9_HUMAN**	**sp|P62701|RS4X_HUMAN**	**93**	**259**	**2091.159**	**698.061**	**3**	**-2.7**	**27.09**	
85	**AKELLGQGLLLR-KLCDNLR-a2-b1**	**sp|Q99613|EIF3C_HUMAN**	**sp|Q14152|EIF3A_HUMAN**	**643**	**196**	**2365.343**	**592.344**	**4**	**-3.5**	**26.92**	
86	**DVKDGK-KFTYR-a3-b1**	**sp|P62269|RS18_HUMAN**	**sp|P62841|RS15_HUMAN**	**91**	**14**	**1511.792**	**378.956**	**4**	**-4.5**	**26.91**	
87	**KQVVNIPSFIVR-LAAKQSSG-a1-b4**	**sp|P46781|RS9_HUMAN**	**sp|P62701|RS4X_HUMAN**	**139**	**259**	**2297.308**	**766.777**	**3**	**-1.3**	**26.63**	
88	**FQQKQIENVLR-LEAMCFDGVKR-a4-b10**	**sp|P20042|IF2B_HUMAN**	**sp|P47813|IF1AX_HUMAN,db|PROT02|IF1A**	**265**	**90**	**2864.453**	**717.121**	**4**	**-5.3**	**26.51**	
89	**AKELLGQGLLLR-TQKTAYQR-a2-b3**	**sp|Q99613|EIF3C_HUMAN**	**sp|O15371|EIF3D_HUMAN**	**643**	**90**	**2442.381**	**611.603**	**4**	**-5.9**	**26.34**	
90	**EGVMVAKK-KFGQGSR-a7-b1**	**sp|P46783|RS10_HUMAN**	**sp|P62273|RS29_HUMAN**	**24**	**13**	**1776.95**	**593.324**	**3**	**-3.3**	**26.04**	
91	**DSDKTDTDWR-YKLLGGLAVR-a4-b2**	**sp|P23588|IF4B_HUMAN,db|PROT04|IF4B_HUM**	**sp|P23396|RS3_HUMAN**	**223**	**108**	**2464.245**	**617.069**	**4**	**-5.9**	**25.79**	
92	**NTKGGDAPAAGEDA-IAGQVAAANKK-a3-b10**	**sp|P62851|RS25_HUMAN**	**sp|P39019|RS19_HUMAN**	**114**	**143**	**2480.243**	**827.756**	**3**	**-3**	**25.45**	
93	**ATVPKTEIR-KAAATPAK-a5-b1**	**sp|P62847|RS24_HUMAN**	**sp|P19338|NUCL_HUMAN**	**37**	**88**	**1908.1**	**478.033**	**4**	**-2.5**	**25.31**	
94	**KGQGGAGAGDDEEED-VTKVLGR-a1-b3**	**sp|P46781|RS9_HUMAN**	**sp|P62857|RS28_HUMAN**	**180**	**16**	**2343.112**	**782.045**	**3**	**-3.1**	**25.22**	
95	**ILKSK-QFQKF-a3-b4**	**sp|P62277|RS13_HUMAN**	**sp|P62280|RS11_HUMAN**	**76**	**157**	**1421.824**	**474.949**	**3**	**-2.8**	**24.74**	
96	**EKGYIDLSK-VAKSNR-a2-b3**	**sp|P05198|IF2A_HUMAN**	**sp|P46782|RS5_HUMAN**	**80**	**201**	**1863**	**466.758**	**4**	**-5.4**	**24.7**	
97	**IDLVKESQAK-NPDMVAGEKR-a5-b9**	**sp|P41091|IF2G_HUMAN**	**sp|P20042|IF2B_HUMAN**	**196**	**199**	**2383.233**	**596.816**	**4**	**-3.8**	**24.51**	
98	**EKNPDMVAGEK-IDLVKESQAK-a2-b5**	**sp|P20042|IF2B_HUMAN**	**sp|P41091|IF2G_HUMAN**	**190**	**196**	**2484.27**	**622.075**	**4**	**-3.4**	**24.4**	

	A	B	C	D	E	F	G	H	I	J	K
99	**MNGKVWDLFPEADK-KAEFR-a4-b1**	**sp|Q99613|EIF3C_HUMAN**	**sp|Q14152|EIF3A_HUMAN**	**764**	**191**	**2436.209**	**813.077**	**3**	**-2.4**	**23.94**	
