# Supplementary material for: Structure of a human cap-dependent 48S translation pre-initiation complex
Source: Nucleic Acids Res. 2018 Feb 1;46(5):2678–89. doi: 10.1093/nar/gky054 (PMC5861459; doi:10.1093/nar/gky054)
Supplement: Supplementary Data [file gky054_supp.zip › nar-01614-r-2017-File010.pdf]

	A	B	C	D	E	F	G	H	I	J	K
1	(B) Supplementary Table S1, listing the intra-molecular Lys-Lys cross-links by DSS in the human 48S complex identified in XL-MS.										
2	Id	Protein1	Protein2	AbsPos1	AbsPos2	Mr	Mz	z	Error_rel[ppm]	Id-Score	Comments
3	MVEKDQDGGR-KLTPQGQR-a4-b1	sp|P39019|RS19_HUMAN	sp|P39019|RS19_HUMAN	115	122	2198.11	733.711	3	-0.7	39.48	high/high data
4	NIGLGFKTPK-FNVLKVTK-a7-b5	sp|P62280|RS11_HUMAN	sp|P62280|RS11_HUMAN	45	144	2159.266	720.763	3	-2.7	39.33	
5	LIKVHLDK-VKLDGSR-a3-b2	sp|P62081|RS7_HUMAN	sp|P62081|RS7_HUMAN	155	147	1876.108	626.377	3	-3.6	39.15	
6	FNVLKVTK-KQFQK-a5-b1	sp|P62280|RS11_HUMAN	sp|P62280|RS11_HUMAN	144	153	1763.032	588.685	3	-1.3	38.68	
7	NKEEAAEYAKLLAK-MKEAK-a10-b2	sp|P62753|RS6_HUMAN	sp|P62753|RS6_HUMAN	211	218	2320.22	581.063	4	-6.5	38.58	
8	KVVGCSCVVVK-DYGKESQAK-a1-b4	sp|P25398|RS12_HUMAN	sp|P25398|RS12_HUMAN	102	116	2396.199	799.741	3	-3.7	38.55	
9	LAAKQSSG-LIYDTKGR-a4-b6	sp|P62701|RS4X_HUMAN	sp|P62701|RS4X_HUMAN	259	106	1863.003	622.009	3	-3.8	38.32	
10	AKELLGQGLLLR-NQEQEKVER-a2-b6	sp|Q99613|EIF3C_HUMAN	sp|Q99613|EIF3C_HUMAN	643	664	2606.433	652.616	4	-2.3	38.22	
11	GYIDLSKR-SINKLIR-a7-b4	sp|P05198|IF2A_HUMAN	sp|P05198|IF2A_HUMAN	87	61	1931.116	483.787	4	-2.1	38.2	
12	LNKCGVISPR-SINNAEKR-a3-b7	sp|P62244|RS15A_HUMAN	sp|P62244|RS15A_HUMAN	71	19	2211.17	553.8	4	-4.2	38.18	
13	CKELGITALHIK-SGMKIGR-a2-b4	sp|P62263|RS14_HUMAN	sp|P62263|RS14_HUMAN	86	125	2267.242	567.818	4	-3.4	38.17	
14	GAVIATELKNNSYK-QKLDSQR-a9-b2	sp|O15371|EIF3D_HUMAN	sp|O15371|EIF3D_HUMAN	426	412	2518.328	840.45	3	-4.5	38.11	
15	NVKGPVR-VTKVLGR-a3-b3	sp|P62857|RS28_HUMAN	sp|P62857|RS28_HUMAN	47	16	1678.019	420.513	4	-3.7	38.08	
16	QYKIPDWFLNR-KADIDLTK-a3-b1	sp|P62269|RS18_HUMAN	sp|P62269|RS18_HUMAN	78	47	2519.332	840.785	3	-4	38.04	
17	FNVLKVTK-KQFQKF-a5-b1	sp|P62280|RS11_HUMAN	sp|P62280|RS11_HUMAN	144	153	1910.095	637.706	3	-4	38.04	
18	AEDKEWMPVTK-LVKDMK-a4-b3	sp|P15880|RS2_HUMAN	sp|P15880|RS2_HUMAN	58	71	2203.116	551.787	4	-4.9	37.98	
19	VLQALEGLKMVEK-LKVPEWVDTVK-a9-b2	sp|P39019|RS19_HUMAN	sp|P39019|RS19_HUMAN	111	29	2907.641	727.918	4	0.5	37.94	
20	SFSKEVEER-TPATKR-a4-b5	sp|Q04637|IF4G1_HUMAN,db|PROT05|IF4G1_H	sp|Q04637|IF4G1_HUMAN,db|PROT05|IF4	1221	1216	1919.985	641.003	3	-5.6	37.92	
21	DYQDNKADVILK-LEAMCFDGVKR-a6-b10	sp|P47813|IF1AX_HUMAN,db|PROT02|IF1AX_H	sp|P47813|IF1AX_HUMAN,db|PROT02|IF1A	122	90	2883.397	721.857	4	-6.2	37.7	
22	VLQALEGLKMVEK-KLTPQGQR-a9-b1	sp|P39019|RS19_HUMAN	sp|P39019|RS19_HUMAN	111	122	2521.429	631.365	4	-0.3	37.61	
23	KVVGCSCVVVK-VDDNKK-a1-b5	sp|P25398|RS12_HUMAN	sp|P25398|RS12_HUMAN	102	83	2089.091	697.372	3	0.1	37.56	
24	GYVKEQFAWR-AMQSLKSR-a4-b6	sp|P46783|RS10_HUMAN	sp|P46783|RS10_HUMAN	59	53	2340.196	781.073	3	-3.9	37.41	
25	QKEMDEAATAEER-LKEELEEAR-a2-b2	sp|Q04637|IF4G1_HUMAN,db|PROT05|IF4G1_H	sp|Q04637|IF4G1_HUMAN,db|PROT05|IF4	899	914	2760.312	921.112	3	-0.3	37.39	
26	TQGTKIASDGLK-NIGKTLVTR-a5-b4	sp|P61247|RS3A_HUMAN	sp|P61247|RS3A_HUMAN	56	46	2356.319	786.448	3	-5.7	37.35	
27	KVPQVSTPTLVEVSR-KVPQVSTPTLVEVSR-a1-b1	sp|P02769|ALBU_BOVIN	sp|P02769|ALBU_BOVIN	437	437	3415.914	854.986	4	-4.4	37.19	contaminant
28	EVPNYKLITPAVVSER-NTKGGDAPAAGEDA-a6-b3	sp|P62851|RS25_HUMAN	sp|P62851|RS25_HUMAN	66	114	3224.609	1075.878	3	-3.4	37.15	
29	NTKGGDAPAAGEDA-GLIKLVSK-a3-b4	sp|P62851|RS25_HUMAN	sp|P62851|RS25_HUMAN	114	98	2267.195	756.739	3	-2.7	37.15	
30	NTKGGDAPAAGEDA-AALQELLSKGLIK-a3-b9	sp|P62851|RS25_HUMAN	sp|P62851|RS25_HUMAN	114	94	2793.467	932.163	3	-3.3	37.12	
31	VPTTEKPTVTVNFR-KLLLNR-a6-b1	sp|Q04637|IF4G1_HUMAN,db|PROT05|IF4G1_H	sp|Q04637|IF4G1_HUMAN,db|PROT05|IF4	865	874	2481.415	621.362	4	-6.9	37.04	
32	TQGTKIASDGLK-VVDPFSKK-a5-b7	sp|P61247|RS3A_HUMAN	sp|P61247|RS3A_HUMAN	56	27	2274.239	759.087	3	-3.6	36.92	
33	LFNLSKEDDVR-KPLNK-a6-b1	sp|P62753|RS6_HUMAN	sp|P62753|RS6_HUMAN	149	160	2071.124	518.789	4	-3.7	36.9	
34	KIFVGTK-LCKVR-a1-b3	sp|P62701|RS4X_HUMAN	sp|P62701|RS4X_HUMAN	128	125	1603.944	401.994	4	-3	36.88	
35	DQKDGYR-KNEGYMR-a3-b1	sp|Q99613|EIF3C_HUMAN	sp|Q99613|EIF3C_HUMAN	889	894	1914.883	639.302	3	-3.3	36.86	
36	CQKEFEK-KLLLNR-a3-b1	sp|Q04637|IF4G1_HUMAN,db|PROT05|IF4G1_H	sp|Q04637|IF4G1_HUMAN,db|PROT05|IF4	882	874	1861.003	466.259	4	-5.5	36.8	
37	DWYDVKAPAMFNIR-ATGDETGAKVER-a6-b9	sp|P61247|RS3A_HUMAN	sp|P61247|RS3A_HUMAN	34	249	3095.494	774.881	4	-2.7	36.79	
38	QPFKDLGNLR-FKQIQR-a4-b2	sp|P55884|EIF3B_HUMAN	sp|P55884|EIF3B_HUMAN	288	366	2143.183	715.402	3	-3.2	36.79	
39	AALQELLSKGLIK-LCKEVPNYK-a9-b3	sp|P62851|RS25_HUMAN	sp|P62851|RS25_HUMAN	94	60	2670.491	668.631	4	-4.4	36.73	
40	FNVLKVTK-QFQKF-a5-b4	sp|P62280|RS11_HUMAN	sp|P62280|RS11_HUMAN	144	157	1782.001	595.008	3	-3.9	36.72	
41	KGQGGAGAGDDEEED-KQVVNIPSFIVR-a1-b1	sp|P46781|RS9_HUMAN	sp|P46781|RS9_HUMAN	180	139	2970.442	991.155	3	-5	36.68	
42	ATGDETGAKVER-NIGKTLVTR-a9-b4	sp|P61247|RS3A_HUMAN	sp|P61247|RS3A_HUMAN	249	46	2371.265	791.429	3	-2.6	36.66	
43	KLFNLSK-KPLNK-a1-b1	sp|P62753|RS6_HUMAN	sp|P62753|RS6_HUMAN	143	160	1584.958	397.247	4	-1.2	36.57	
44	STPKEDDSSASTSQSTR-AASIFGGAKPVDTAAR-a4-b9	sp|P23588|IF4B_HUMAN,db|PROT04|IF4B_HUM	sp|P23588|IF4B_HUMAN,db|PROT04|IF4B_	372	394	3451.65	1151.558	3	-5.8	36.49	
45	ATGDETGAKVER-TQGTKIASDGLK-a9-b5	sp|P61247|RS3A_HUMAN	sp|P61247|RS3A_HUMAN	249	56	2588.323	648.088	4	-2.7	36.49	
46	TELGAKAFK-LEQIKK-a6-b5	sp|Q14152|EIF3A_HUMAN	sp|Q14152|EIF3A_HUMAN	651	644	1859.067	465.775	4	-5.4	36.45	
47	DHASIQMNVAEVDKVTGR-ADGIVSKNF-a14-b7	sp|P63220|RS21_HUMAN	sp|P63220|RS21_HUMAN	41	81	3056.515	765.137	4	-2.8	36.35	
48	VETFSGVYKK-VKLDGSR-a9-b2	sp|P62081|RS7_HUMAN	sp|P62081|RS7_HUMAN	178	147	2068.116	690.38	3	-2.3	36.31	
49	IASDGLKGR-NIGKTLVTR-a7-b4	sp|P61247|RS3A_HUMAN	sp|P61247|RS3A_HUMAN	63	46	2054.174	514.551	4	-5.1	36.23	
50	KVPQVSTPTLVEVSR-SLGKVGTR-a1-b4	sp|P02769|ALBU_BOVIN	sp|P02769|ALBU_BOVIN	437	455	2593.469	649.375	4	-4.3	36.21	contaminant
51	GNKPWISLPR-LAAKQSSG-a3-b4	sp|P62701|RS4X_HUMAN	sp|P62701|RS4X_HUMAN	233	259	2065.121	689.382	3	-5.3	36.2	
52	SPDTILQKDTR-TGFQAVTGKR-a8-b9	sp|P20042|IF2B_HUMAN	sp|P20042|IF2B_HUMAN	293	324	2474.311	825.778	3	-0.8	36.1	
53	VKAEPFIK-ELAKEIR-a2-b4	sp|P55010|IF5_HUMAN,db|PROT06|IF5_HUMA	sp|P55010|IF5_HUMAN,db|PROT06|IF5_HU	394	389	1926.106	643.043	3	-6.7	36.1	
54	DTGKTPVEPEVAIHR-NLKVK-a4-b3	sp|P60866|RS20_HUMAN	sp|P60866|RS20_HUMAN	8	49	2386.314	478.271	5	-3.2	36.09	
55	DVTKCLEEFELLGK-NAMKIR-a4-b4	sp|Q99613|EIF3C_HUMAN	sp|Q99613|EIF3C_HUMAN	78	72	2549.319	850.781	3	-1.6	36.04	
56	AASIFGGAKPVDTAAR-LNLKPR-a9-b4	sp|P23588|IF4B_HUMAN,db|PROT04|IF4B_HUM	sp|P23588|IF4B_HUMAN,db|PROT04|IF4B_	394	366	2408.347	603.095	4	-3	36.03	
57	LMELHGEGSSSGKATGDETGAK-TQGTKIASDGLK-a13-b5	sp|P61247|RS3A_HUMAN	sp|P61247|RS3A_HUMAN	240	56	3516.711	880.186	4	-4	36.01	
58	TPKEAIEGTYIDK-FNVLKVTK-a3-b5	sp|P62280|RS11_HUMAN	sp|P62280|RS11_HUMAN	48	144	2549.388	638.355	4	-4.6	36	
59	MNVLADALKSINNAEK-LNKCGVISPR-a9-b3	sp|P62244|RS15A_HUMAN	sp|P62244|RS15A_HUMAN	12	71	3010.578	753.652	4	-5.6	35.99	
60	AASIFGGAKPVDTAAR-EQEKLQR-a9-b4	sp|P23588|IF4B_HUMAN,db|PROT04|IF4B_HUM	sp|P23588|IF4B_HUMAN,db|PROT04|IF4B_	394	415	2598.37	867.131	3	-2.4	35.94	
61	EIKDILIQYDR-YVDEASKK-a3-b7	sp|P62249|RS16_HUMAN	sp|P62249|RS16_HUMAN	109	105	2481.287	828.103	3	-5.5	35.89	
62	LSQSKASK-IFEQKDR-a5-b5	sp|P55884|EIF3B_HUMAN	sp|P55884|EIF3B_HUMAN	741	734	1920.027	481.015	4	-2.4	35.79	
63	DIEKACQSIYPLHDVFVR-KMMEIMTR-a4-b1	sp|P61247|RS3A_HUMAN	sp|P61247|RS3A_HUMAN	199	167	3365.655	674.139	5	-2.8	35.78	
64	IYKLDDPSCPRPECYR-GNFKLVR-a3-b4	sp|P41091|IF2G_HUMAN	sp|P41091|IF2G_HUMAN	90	125	3038.501	760.633	4	-3	35.77	
65	AAGTKK-QFQKF-a5-b4	sp|P62280|RS11_HUMAN	sp|P62280|RS11_HUMAN	152	157	1408.766	470.597	3	-3.8	35.77	
66	AASIFGGAKPVDTAAR-DSDKTDTDWR-a9-b4	sp|P23588|IF4B_HUMAN,db|PROT04|IF4B_HUM	sp|P23588|IF4B_HUMAN,db|PROT04|IF4B_	394	223	2906.403	969.809	3	-0.7	35.77	
67	GVTIKPTVDDD-LMCKPIFSK-a5-b4	sp|P41091|IF2G_HUMAN	sp|P41091|IF2G_HUMAN	466	312	2419.232	807.419	3	-2.3	35.73	
68	KTYVTPR-VKFTLAK-a1-b2	sp|P46781|RS9_HUMAN	sp|P46781|RS9_HUMAN	11	47	1807.057	452.772	4	-2.2	35.7	
69	YELGRPAANTKIGPR-TKTLVK-a11-b2	sp|P62241|RS8_HUMAN	sp|P62241|RS8_HUMAN	37	94	2468.405	494.689	5	-2.7	35.6	
70	VQPIKLAR-NVKGPVR-a5-b3	sp|P62857|RS28_HUMAN	sp|P62857|RS28_HUMAN	10	47	1830.111	458.536	4	-5	35.57	
71	LNNLVLFDKATYDK-LCKEVPNYK-a9-b3	sp|P62851|RS25_HUMAN	sp|P62851|RS25_HUMAN	52	60	2940.521	736.138	4	-3.2	35.44	
72	STPKEDDSSASTSQSTR-DSDKTDTDWR-a4-b4	sp|P23588|IF4B_HUMAN,db|PROT04|IF4B_HUM	sp|P23588|IF4B_HUMAN,db|PROT04|IF4B_	372	223	3158.364	790.599	4	-3.7	35.34	
73	GDDSFGDKYR-QLDEPKLER-a8-b6	sp|P23588|IF4B_HUMAN,db|PROT04|IF4B_HUM	sp|P23588|IF4B_HUMAN,db|PROT04|IF4B_	252	424	2423.153	606.796	4	-3.1	35.17	
74	AASIFGGAKPVDTAAR-QLDEPKLER-a9-b6	sp|P23588|IF4B_HUMAN,db|PROT04|IF4B_HUM	sp|P23588|IF4B_HUMAN,db|PROT04|IF4B_	394	424	2795.476	932.833	3	-2.2	35.08	
75	FVNVVPTFGKK-KGPNANS-a10-b1	sp|P62861|RS30_HUMAN	sp|P62861|RS30_HUMAN	51	53	2059.103	687.376	3	-3.4	35.08	
76	LFCVGFTKK-MCSMVKK-a8-b6	sp|P61247|RS3A_HUMAN	sp|P61247|RS3A_HUMAN	144	115	2119.062	530.773	4	-4	35.03	
77	VTKCCTESLVNR-ALKAWSVAR-a3-b3	sp|P02769|ALBU_BOVIN	sp|P02769|ALBU_BOVIN	498	235	2604.349	652.095	4	-1	34.99	contaminant
78	LMELHGEGSSSGKATGDETGAK-EVQTNDLKEVVNK-a13-b8	sp|P61247|RS3A_HUMAN	sp|P61247|RS3A_HUMAN	240	182	3813.851	763.778	5	-1.9	34.98	
79	EDGQEYAQVIKMLGNGR-LEAMCFDGVKR-a11-b10	sp|P47813|IF1AX_HUMAN,db|PROT02|IF1AX_H	sp|P47813|IF1AX_HUMAN,db|PROT02|IF1A	74	90	3369.6	843.408	4	-4.8	34.94	
80	DSDKTDTDWR-QLDEPKLER-a4-b6	sp|P23588|IF4B_HUMAN,db|PROT04|IF4B_HUM	sp|P23588|IF4B_HUMAN,db|PROT04|IF4B_	223	424	2502.179	626.552	4	-3.5	34.9	
81	LITEDVQGKNCLTNFHGMDLTR-TQGTKIASDGLK-a9-b5	sp|P61247|RS3A_HUMAN	sp|P61247|RS3A_HUMAN	94	56	3916.958	980.247	4	-2.2	34.88	
82	AGLNCSTENMPIKINLIAPPR-LTPQAVKIR-a13-b7	sp|P05198|IF2A_HUMAN	sp|P05198|IF2A_HUMAN	226	190	3470.907	868.735	4	-0.9	34.85	
83	DWYDVKAPAMFNIR-NIGKTLVTR-a6-b4	sp|P61247|RS3A_HUMAN	sp|P61247|RS3A_HUMAN	34	46	2863.499	716.883	4	-2.3	34.81	
84	SFDVNKPGCEVDDLK-FKNELER-a6-b2	sp|P41091|IF2G_HUMAN	sp|P41091|IF2G_HUMAN	266	70	2794.335	932.453	3	-4.7	34.77	
85	LITEDVQGKNCLTNFHGMDLTR-NIGKTLVTR-a9-b4	sp|P61247|RS3A_HUMAN	sp|P61247|RS3A_HUMAN	94	46	3699.896	740.987	5	-3	34.7	
86	LDQELKLIGEYGLR-KTYVTPR-a6-b1	sp|P46781|RS9_HUMAN	sp|P46781|RS9_HUMAN	30	11	2647.443	662.869	4	-5.8	34.66	
87	STPKEDDSSASTSQSTR-QLDEPKLER-a4-b6	sp|P23588|IF4B_HUMAN,db|PROT04|IF4B_HUM	sp|P23588|IF4B_HUMAN,db|PROT04|IF4B_	372	424	3047.448	762.87	4	-1.5	34.53	
88	QLDEPKLER-EQEKLQR-a6-b4	sp|P23588|IF4B_HUMAN,db|PROT04|IF4B_HUM	sp|P23588|IF4B_HUMAN,db|PROT04|IF4B_	424	415	2194.155	549.547	4	-2.1	34.37	
89	LMELHGEGSSSGKATGDETGAK-NIGKTLVTR-a13-b4	sp|P61247|RS3A_HUMAN	sp|P61247|RS3A_HUMAN	240	46	3299.666	825.924	4	-0.3	34.36	
90	LIPDSIGKDIEK-LFCVGFTKK-a8-b8	sp|P61247|RS3A_HUMAN	sp|P61247|RS3A_HUMAN	195	144	2563.388	641.855	4	-3.4	34.15	
91	ATGDETGAKVER-IASDGLKGR-a9-b7	sp|P61247|RS3A_HUMAN	sp|P61247|RS3A_HUMAN	249	63	2286.177	572.552	4	-2	34.11	
92	EVQTNDLKEVVNK-NIGKTLVTR-a8-b4	sp|P61247|RS3A_HUMAN	sp|P61247|RS3A_HUMAN	182	46	2653.46	664.373	4	-1.8	34.08	
93	RGVTIKPTVDDD-LMCKPIFSK-a6-b4	sp|P41091|IF2G_HUMAN	sp|P41091|IF2G_HUMAN	466	312	2575.32	859.448	3	-7.2	33.77	
94	EVQTNDLKEVVNK-ATGDETGAKVER-a8-b9	sp|P61247|RS3A_HUMAN	sp|P61247|RS3A_HUMAN	182	249	2885.464	962.829	3	0.8	33.58	
95	FNGQFKTYAICGAIR-KCSASNR-a6-b1	sp|P63220|RS21_HUMAN	sp|P63220|RS21_HUMAN	51	16	2704.316	677.087	4	-2	33.53	
96	VWDLFPEADKVR-KIQEESLR-a10-b1	sp|Q99613|EIF3C_HUMAN	sp|Q99613|EIF3C_HUMAN	774	782	2613.367	654.35	4	-4.9	33.33	
97	KEEGVIDSSDK-VNILFDFVKK-a1-b9	sp|P55010|IF5_HUMAN,db|PROT06|IF5_HUMA	sp|P55010|IF5_HUMAN,db|PROT06|IF5_HU	271	268	2565.356	642.347	4	-0.6	32.98	
98	DPSQIDNNEPYMKIPCNDSK-QLALLKTNSAVR-a13-b6	sp|Q13347|EIF3I_HUMAN	sp|Q13347|EIF3I_HUMAN	141	91	3814.877	954.727	4	-2.6	32.95	
99	VDVADQAQDKDRDDR-DSDKTDTDWR-a10-b4	sp|P23588|IF4B_HUMAN,db|PROT04|IF4B_HUM	sp|P23588|IF4B_HUMAN,db|PROT04|IF4B_	206	223	3120.377	781.102	4	-3.2	32.91	
100	STPKEDDSSASTSQSTR-RGDDSFGDKYR-a4-b9	sp|P23588|IF4B_HUMAN,db|PROT04|IF4B_HUM	sp|P23588|IF4B_HUMAN,db|PROT04|IF4B_	372	252	3235.442	809.868	4	-2.4	32.22	
101	LMELHGEGSSSGKATGDETGAK-DWYDVKAPAMFNIR-a13-b6	sp|P61247|RS3A_HUMAN	sp|P61247|RS3A_HUMAN	240	34	4023.887	805.785	5	-2.8	31.48	
102	RFVNVVPTFGKK-KGPNANS-a11-b1	sp|P62861|RS30_HUMAN	sp|P62861|RS30_HUMAN	51	53	2215.211	739.411	3	-0.2	31.25	
103	QLDEPKLER-LQKEQEK-a6-b3	sp|P23588|IF4B_HUMAN,db|PROT04|IF4B_HUM	sp|P23588|IF4B_HUMAN,db|PROT04|IF4B_	424	411	2166.151	542.546	4	-0.9	30.63	
104											
105	NIGLGFKTPK-FNVLKVTK-a7-b5	sp|P62280|RS11_HUMAN	sp|P62280|RS11_HUMAN	45	144	2159.263	540.824	4	-4	34.3	high/low data
106	SFSKEVEER-TPATKR-a4-b5	sp|Q04637|IF4G1_HUMAN,db|PROT05|IF4G1_H	sp|Q04637|IF4G1_HUMAN,db|PROT05|IF4	1221	1216	1919.988	641.004	3	-3.7	34.15	
107	FNVLKVTK-AAGTKK-a5-b5	sp|P62280|RS11_HUMAN	sp|P62280|RS11_HUMAN	144	152	1659.984	416.004	4	-4.9	33.08	
108	VQPIKLAR-NVKGPVR-a5-b3	sp|P62857|RS28_HUMAN	sp|P62857|RS28_HUMAN	10	47	1830.115	458.536	4	-3.1	32.79	
109	KVVGCSCVVVK-DYGKESQAK-a1-b4	sp|P25398|RS12_HUMAN	sp|P25398|RS12_HUMAN	102	116	2396.198	600.057	4	-4	32.72	
110	CASIQKFGER-ALKAWSVAR-a6-b3	sp|P02769|ALBU_BOVIN	sp|P02769|ALBU_BOVIN	228	235	2333.22	584.313	4	-4.7	32.01	contaminant
111	AAGTKK-QFQKF-a5-b4	sp|P62280|RS11_HUMAN	sp|P62280|RS11_HUMAN	152	157	1408.766	470.596	3	-4.1	31.86	

	A	B	C	D	E	F	G	H	I	J	K
112	NVKGPVR-VTKVLGR-a3-b3	sp|P62857|RS28_HUMAN	sp|P62857|RS28_HUMAN	47	16	1678.019	420.513	4	-3.9	31.66	
113	LNKCGVISPR-SINNAEKR-a3-b7	sp|P62244|RS15A_HUMAN	sp|P62244|RS15A_HUMAN	71	19	2211.171	553.8	4	-4	31.6	
114	VDVAVLKAR-AKLLQK-a7-b2	sp|Q04637|IF4G1_HUMAN,db|PROT05|IF4G1_H	sp|Q04637|IF4G1_HUMAN,db|PROT05|IF4	1538	1542	1807.119	452.788	4	-5.8	31.02	
115	TKNETDEDGWTTVR-AEKDR-a2-b3	sp|Q14152|EIF3A_HUMAN	sp|Q14152|EIF3A_HUMAN	1369	1360	2406.12	602.538	4	-4.3	30.89	
116	LIKVHLDK-VKLDGSR-a3-b2	sp|P62081|RS7_HUMAN	sp|P62081|RS7_HUMAN	155	147	1876.107	470.035	4	-4	30.42	
117	KVVGCSCVVVK-VDDNKK-a1-b5	sp|P25398|RS12_HUMAN	sp|P25398|RS12_HUMAN	102	83	2089.086	697.37	3	-2.5	30.07	
118	TKTPGPGAQSALR-SGMKIGR-a2-b4	sp|P62263|RS14_HUMAN	sp|P62263|RS14_HUMAN	106	125	2168.164	543.049	4	-4.5	30	
119	GYIDLSKR-SINKLIR-a7-b4	sp|P05198|IF2A_HUMAN	sp|P05198|IF2A_HUMAN	87	61	1931.113	644.712	3	-3.8	29.92	
120	QLDEPKLER-LQKEQEK-a6-b3	sp|P23588|IF4B_HUMAN,db|PROT04|IF4B_HUN	sp|P23588|IF4B_HUMAN,db|PROT04|IF4B_	424	411	2166.14	542.543	4	-6.3	29.89	
121	DVTKCLEEFELLGK-NAMKIR-a4-b4	sp|Q99613|EIF3C_HUMAN	sp|Q99613|EIF3C_HUMAN	78	72	2549.314	850.779	3	-3.9	29.6	
122	FNVLKVTK-KQFQKF-a5-b1	sp|P62280|RS11_HUMAN	sp|P62280|RS11_HUMAN	144	153	1910.097	637.707	3	-3.1	29.57	
123	QYKIPDWFLNR-KADIDLTK-a3-b1	sp|P62269|RS18_HUMAN	sp|P62269|RS18_HUMAN	78	47	2519.335	630.842	4	-2.9	29.3	
124	ELNITAAKEIEVGGGR-KLTGK-a8-b1	sp|P62081|RS7_HUMAN	sp|P62081|RS7_HUMAN	49	179	2339.297	585.832	4	-3.8	29.25	
125	GYVKEQFAWR-AMQSLKSR-a4-b6	sp|P46783|RS10_HUMAN	sp|P46783|RS10_HUMAN	59	53	2340.197	586.057	4	-3.5	28.85	
126	VLQALEGLKMVEK-KLTPQGQR-a9-b1	sp|P39019|RS19_HUMAN	sp|P39019|RS19_HUMAN	111	122	2521.422	631.363	4	-3.1	28.74	
127	VQPIKLAR-VTKVLGR-a5-b3	sp|P62857|RS28_HUMAN	sp|P62857|RS28_HUMAN	10	16	1833.15	459.295	4	-3.6	28.64	
128	LFNLSKEDDVR-KPLNK-a6-b1	sp|P62753|RS6_HUMAN	sp|P62753|RS6_HUMAN	149	160	2071.122	518.788	4	-4.4	28.28	
129	CKELGITALHIK-SGMKIGR-a2-b4	sp|P62263|RS14_HUMAN	sp|P62263|RS14_HUMAN	86	125	2267.239	567.817	4	-4.7	28.25	
130	KIDYFER-LKNQEK-a1-b2	sp|Q14152|EIF3A_HUMAN	sp|Q14152|EIF3A_HUMAN	694	689	1865.98	467.503	4	-4.8	28.12	
131	AEDKEWMPVTK-LVKDMK-a4-b3	sp|P15880|RS2_HUMAN	sp|P15880|RS2_HUMAN	58	71	2203.117	551.787	4	-4.3	27.89	
132	KIFVGTK-LCKVR-a1-b3	sp|P62701|RS4X_HUMAN	sp|P62701|RS4X_HUMAN	128	125	1603.942	401.993	4	-4.2	27.86	
133	FNVLKVTK-KQFQK-a5-b1	sp|P62280|RS11_HUMAN	sp|P62280|RS11_HUMAN	144	153	1763.027	588.684	3	-4.2	27.66	
134	TQGTKIASDGLK-NIGKTLVTR-a5-b4	sp|P61247|RS3A_HUMAN	sp|P61247|RS3A_HUMAN	56	46	2356.323	786.449	3	-3.9	27.24	
135	MVEKDQDGGR-KLTPQGQR-a4-b1	sp|P39019|RS19_HUMAN	sp|P39019|RS19_HUMAN	115	122	2198.102	550.533	4	-4.3	27.21	
136	LSQSKASK-IFEQKDR-a5-b5	sp|P55884|EIF3B_HUMAN	sp|P55884|EIF3B_HUMAN	741	734	1920.022	641.015	3	-5.2	26.71	
137	GAVIATELKNNSYK-QKLDSQR-a9-b2	sp|O15371|EIF3D_HUMAN	sp|O15371|EIF3D_HUMAN	426	412	2518.329	840.451	3	-4	26.58	
138	NTKGGDAPAAGEDA-GLIKLVSK-a3-b4	sp|P62851|RS25_HUMAN	sp|P62851|RS25_HUMAN	114	98	2267.196	756.74	3	-2.1	26.35	
139	SKGIAYIEFK-LVSKDGK-a2-b4	sp|P19338|NUCL_HUMAN	sp|P19338|NUCL_HUMAN	429	424	2038.128	510.54	4	-3.4	26.24	
140	CCTKPESER-SLGKVGTR-a4-b4	sp|P02769|ALBU_BOVIN	sp|P02769|ALBU_BOVIN	463	455	2120.027	531.015	4	-3.8	26.07	
141	EVPNYKLITPAVVSER-NTKGGDAPAAGEDA-a6-b3	sp|P62851|RS25_HUMAN	sp|P62851|RS25_HUMAN	66	114	3224.608	1075.877	3	-3.6	25.91	
142	SPDTILQKDTR-TGFQAVTGKR-a8-b9	sp|P20042|IF2B_HUMAN	sp|P20042|IF2B_HUMAN	293	324	2474.303	825.776	3	-3.8	25.87	
143	EIKDILIQYDR-YVDEASKK-a3-b7	sp|P62249|RS16_HUMAN	sp|P62249|RS16_HUMAN	109	105	2481.292	621.331	4	-3.3	25.86	
144	LKEELEEAR-KTSSR-a2-b1	sp|Q04637|IF4G1_HUMAN,db|PROT05|IF4G1_H	sp|Q04637|IF4G1_HUMAN,db|PROT05|IF4	914	1003	1830.962	611.329	3	-3.6	25.84	
145	KQFQKF-MKMQR-a1-b2	sp|P62280|RS11_HUMAN	sp|P62280|RS11_HUMAN	153	81	1654.861	414.723	4	-4.6	25.7	
146	LFKPPQPPAR-NTSKQQQQK-a3-b4	sp|O15372|EIF3H_HUMAN	sp|O15372|EIF3H_HUMAN	306	269	2376.283	595.079	4	-3.6	25.7	
147	AASIFGGAKPVDTAAR-DSDKTDTDWR-a9-b4	sp|P23588|IF4B_HUMAN,db|PROT04|IF4B_HUN	sp|P23588|IF4B_HUMAN,db|PROT04|IF4B_	394	223	2906.394	727.606	4	-3.5	25.69	
148	LMELHGEGSSSGKATGDETGAK-DWYDVKAPAMFNIR-a13-b6	sp|P61247|RS3A_HUMAN	sp|P61247|RS3A_HUMAN	240	34	4023.87	805.782	5	-7	25.53	
149	VPTTEKPTVTVNFR-KLLLNR-a6-b1	sp|Q04637|IF4G1_HUMAN,db|PROT05|IF4G1_H	sp|Q04637|IF4G1_HUMAN,db|PROT05|IF4	865	874	2481.423	621.364	4	-3.4	25.32	
150	TPKEAIEGTYIDK-FNVLKVTK-a3-b5	sp|P62280|RS11_HUMAN	sp|P62280|RS11_HUMAN	48	144	2549.389	850.804	3	-3.9	25.15	
151	DYQDNKADVILK-LEAMCFDGVKR-a6-b10	sp|P47813|IF1AX_HUMAN,db|PROT02|IF1AX_H	sp|P47813|IF1AX_HUMAN,db|PROT02|IF1	122	90	2883.407	962.143	3	-2.8	25.02	
152	DWYDVKAPAMFNIR-ATGDETGAKVER-a6-b9	sp|P61247|RS3A_HUMAN	sp|P61247|RS3A_HUMAN	34	249	3095.488	774.88	4	-4.5	24.94	
153	DIEKACQSIYPLHDVFVR-KMMEIMTR-a4-b1	sp|P61247|RS3A_HUMAN	sp|P61247|RS3A_HUMAN	199	167	3365.646	674.137	5	-5.6	24.61	
154	VTKCCTESLVNR-SLGKVGTR-a3-b4	sp|P02769|ALBU_BOVIN	sp|P02769|ALBU_BOVIN	498	455	2420.243	606.068	4	-3.7	24.57	contaminant
155	KVPQVSTPTLVEVSR-ALKAWSVAR-a1-b3	sp|P02769|ALBU_BOVIN	sp|P02769|ALBU_BOVIN	437	235	2777.565	695.399	4	-5.6	24.44	contaminant
156	NTKGGDAPAAGEDA-AALQELLSKGLIK-a3-b9	sp|P62851|RS25_HUMAN	sp|P62851|RS25_HUMAN	114	94	2793.47	932.165	3	-2.1	24.39	
157	AKELLGQGLLLR-NQEQEKVER-a2-b6	sp|Q99613|EIF3C_HUMAN	sp|Q99613|EIF3C_HUMAN	643	664	2606.432	652.616	4	-2.6	24.39	
158	FNGQFKTYAICGAIR-KCSASNR-a6-b1	sp|P63220|RS21_HUMAN	sp|P63220|RS21_HUMAN	51	16	2704.311	677.085	4	-4	24.37	
159	DQKDGYR-KNEGYMR-a3-b1	sp|Q99613|EIF3C_HUMAN	sp|Q99613|EIF3C_HUMAN	889	894	1914.881	479.728	4	-4.6	24.31	
160	VWDLFPEADKVR-KIQEESLR-a10-b1	sp|Q99613|EIF3C_HUMAN	sp|Q99613|EIF3C_HUMAN	774	782	2613.374	654.351	4	-2.6	24.25	
161	KQKEMDEAATAEER-LKEELEEAR-a3-b2	sp|Q04637|IF4G1_HUMAN,db|PROT05|IF4G1_H	sp|Q04637|IF4G1_HUMAN,db|PROT05|IF4	899	914	2888.394	723.106	4	-4.8	24.2	
162	TSIFWNDVKDPVSIEER-LLQKNNK-a9-b4	sp|P55884|EIF3B_HUMAN	sp|P55884|EIF3B_HUMAN	324	693	3028.577	758.152	4	-3.3	24.19	
163	ATGDETGAKVER-IASDGLKGR-a9-b7	sp|P61247|RS3A_HUMAN	sp|P61247|RS3A_HUMAN	249	63	2286.173	572.551	4	-3.7	24.18	
164	ELNITAAKEIEVGGGRK-KLTGK-a8-b1	sp|P62081|RS7_HUMAN	sp|P62081|RS7_HUMAN	49	179	2467.393	494.486	5	-3.4	23.85	
165	YYKVDENGK-VKLAVLK-a3-b2	sp|P62979|RS27A_HUMAN	sp|P62979|RS27A_HUMAN	107	99	2022.134	506.541	4	-2.8	23.79	
166	VDVADQAQDKDRDDR-DSDKTDTDWR-a10-b4	sp|P23588|IF4B_HUMAN,db|PROT04|IF4B_HUN	sp|P23588|IF4B_HUMAN,db|PROT04|IF4B_	206	223	3120.373	781.101	4	-4.3	23.79	
167	GNKPWISLPR-RLAAKQSSG-a3-b5	sp|P62701|RS4X_HUMAN	sp|P62701|RS4X_HUMAN	233	259	2221.224	556.314	4	-4.2	23.73	
168	LITEDVQGKNCLTNFHGMDLTR-NIGKTLVTR-a9-b4	sp|P61247|RS3A_HUMAN	sp|P61247|RS3A_HUMAN	94	46	3699.895	740.987	5	-3.4	23.47	
169	AVTTPGKK-VVPVKAK-a7-b5	sp|P19338|NUCL_HUMAN	sp|P19338|NUCL_HUMAN	109	228	1678.034	420.516	4	-3.4	23.4	
170	DLEKWQNNLLPSR-LNKCGVISPR-a4-b3	sp|P62244|RS15A_HUMAN	sp|P62244|RS15A_HUMAN	88	71	2892.516	724.137	4	-4.2	23.33	
171	QPFKDLGNLR-FKQIQR-a4-b2	sp|P55884|EIF3B_HUMAN	sp|P55884|EIF3B_HUMAN	288	366	2143.184	536.804	4	-2.9	23.3	
172	STPKEDDSSASTSQSTR-AASIFGGAKPVDTAAR-a4-b9	sp|P23588|IF4B_HUMAN,db|PROT04|IF4B_HUN	sp|P23588|IF4B_HUMAN,db|PROT04|IF4B_	372	394	3451.662	863.923	4	-2.5	23.15	
173	LCVLHEKTPVSEK-VTKCCTESLVNR-a7-b3	sp|P02769|ALBU_BOVIN	sp|P02769|ALBU_BOVIN	489	498	3142.577	786.652	4	-1.7	23.03	contaminant
174	VETFSGVYKK-VKLDGSR-a9-b2	sp|P62081|RS7_HUMAN	sp|P62081|RS7_HUMAN	178	147	2068.113	518.036	4	-3.8	22.83	
175	LKVPEWVDTVK-MVEKDQDGGR-a2-b4	sp|P39019|RS19_HUMAN	sp|P39019|RS19_HUMAN	29	115	2584.31	647.085	4	-4	22.82	
176	EVQTNDLKEVVNK-ATGDETGAKVER-a8-b9	sp|P61247|RS3A_HUMAN	sp|P61247|RS3A_HUMAN	182	249	2885.45	722.37	4	-4	22.79	
177	ASKELVER-LLQKNNK-a3-b4	sp|P55884|EIF3B_HUMAN	sp|P55884|EIF3B_HUMAN	744	693	1925.086	482.279	4	-4.5	22.74	
178	ATGDETGAKVER-TQGTKIASDGLK-a9-b5	sp|P61247|RS3A_HUMAN	sp|P61247|RS3A_HUMAN	249	56	2588.317	648.087	4	-4.6	22.73	
179	AASIFGGAKPVDTAAR-EQEKLQR-a9-b4	sp|P23588|IF4B_HUMAN,db|PROT04|IF4B_HUN	sp|P23588|IF4B_HUMAN,db|PROT04|IF4B_	394	415	2598.369	867.131	3	-2.9	22.71	
180	ATGDETGAKVER-NIGKTLVTR-a9-b4	sp|P61247|RS3A_HUMAN	sp|P61247|RS3A_HUMAN	249	46	2371.261	593.823	4	-4.2	22.69	
181	LMELHGEGSSSGKATGDETGAK-TQGTKIASDGLK-a13-b5	sp|P61247|RS3A_HUMAN	sp|P61247|RS3A_HUMAN	240	56	3516.711	880.186	4	-4	22.68	
182	RGVTIKPTVDDD-LMCKPIFSK-a6-b4	sp|P41091|IF2G_HUMAN	sp|P41091|IF2G_HUMAN	466	312	2575.328	644.84	4	-4.3	22.56	
183	LITEDVQGKNCLTNFHGMDLTR-TQGTKIASDGLK-a9-b5	sp|P61247|RS3A_HUMAN	sp|P61247|RS3A_HUMAN	94	56	3916.951	784.398	5	-4	22.44	
184	LMELHGEGSSSGKATGDETGAK-EVQTNDLKEVVNK-a13-b8	sp|P61247|RS3A_HUMAN	sp|P61247|RS3A_HUMAN	240	182	3813.841	763.776	5	-4.4	22.36	
185	VLQALEGLKMVEK-LKVPEWVDTVK-a9-b2	sp|P39019|RS19_HUMAN	sp|P39019|RS19_HUMAN	111	29	2907.63	727.915	4	-3.4	22.21	
186	STPKEDDSSASTSQSTR-QLDEPKLER-a4-b6	sp|P23588|IF4B_HUMAN,db|PROT04|IF4B_HUN	sp|P23588|IF4B_HUMAN,db|PROT04|IF4B_	372	424	3047.442	762.868	4	-3.8	22.12	
187	DWYDVKAPAMFNIR-NIGKTLVTR-a6-b4	sp|P61247|RS3A_HUMAN	sp|P61247|RS3A_HUMAN	34	46	2863.493	716.881	4	-4.3	22	
188	QKEMDEAATAEER-LKEELEEAR-a2-b2	sp|Q04637|IF4G1_HUMAN,db|PROT05|IF4G1_H	sp|Q04637|IF4G1_HUMAN,db|PROT05|IF4	899	914	2760.305	921.11	3	-2.6	21.86	
189	DHASIQMNVAEVDKVTGR-ADGIVSKNF-a14-b7	sp|P63220|RS21_HUMAN	sp|P63220|RS21_HUMAN	41	81	3056.513	765.136	4	-3.4	21.85	
190	DIEKACQSIYPLHDVFVR-ATGDETGAKVER-a4-b9	sp|P61247|RS3A_HUMAN	sp|P61247|RS3A_HUMAN	199	249	3559.747	712.957	5	-4.1	21.72	
191	GNKPWISLPR-LAAKQSSG-a3-b4	sp|P62701|RS4X_HUMAN	sp|P62701|RS4X_HUMAN	233	259	2065.122	689.382	3	-5.1	21.61	
192	LMELHGEGSSSGKATGDETGAK-NIGKTLVTR-a13-b4	sp|P61247|RS3A_HUMAN	sp|P61247|RS3A_HUMAN	240	46	3299.658	660.939	5	-2.7	21.54	
193	KVVGCSCVVVK-KLGEWVGLCK-a1-b1	sp|P25398|RS12_HUMAN	sp|P25398|RS12_HUMAN	102	84	2560.351	641.096	4	-2.8	21.53	
194	LNNLVLFDKATYDK-LCKEVPNYK-a9-b3	sp|P62851|RS25_HUMAN	sp|P62851|RS25_HUMAN	52	60	2940.524	736.139	4	-2.2	21.52	
195	LVTDLTKVHK-ALKAWSVAR-a7-b3	sp|P02769|ALBU_BOVIN	sp|P02769|ALBU_BOVIN	263	235	2291.33	573.84	4	-2.8	21.21	contaminant
196	KGQGGAGAGDDEEED-KQVVNIPSFIVR-a1-b1	sp|P46781|RS9_HUMAN	sp|P46781|RS9_HUMAN	180	139	2970.453	991.159	3	-1.4	21.12	
197	ATGDETGAKVER-KPKFELGK-a9-b1	sp|P61247|RS3A_HUMAN	sp|P61247|RS3A_HUMAN	249	220	2316.219	580.063	4	-5.7	20.88	
198	VTGGMKVK-SGMKIGR-a6-b4	sp|P62263|RS14_HUMAN	sp|P62263|RS14_HUMAN	61	125	1703.937	568.987	3	-3.4	20.78	
199	LAKEYEATLEECCAK-SLGKVGTR-a3-b4	sp|P02769|ALBU_BOVIN	sp|P02769|ALBU_BOVIN	374	455	2768.356	693.097	4	-5.9	20.77	contaminant
200	KEEGVIDSSDK-VNILFDFVKK-a1-b9	sp|P55010|IF5_HUMAN,db|PROT06|IF5_HUMA	sp|P55010|IF5_HUMAN,db|PROT06|IF5_HU	271	268	2565.348	642.345	4	-3.8	20.47	
201	AALQELLSKGLIK-LCKEVPNYK-a9-b3	sp|P62851|RS25_HUMAN	sp|P62851|RS25_HUMAN	94	60	2670.495	668.632	4	-2.9	20.3	
202	NTKGGDAPAAGEDA-LCKEVPNYK-a3-b3	sp|P62851|RS25_HUMAN	sp|P62851|RS25_HUMAN	114	60	2560.203	854.409	3	-3.4	20.21	
203	KVPQVSTPTLVEVSR-SLGKVGTR-a1-b4	sp|P02769|ALBU_BOVIN	sp|P02769|ALBU_BOVIN	437	455	2593.472	649.376	4	-3.2	19.67	contaminant
204	VTKCCTESLVNR-ALKAWSVAR-a3-b3	sp|P02769|ALBU_BOVIN	sp|P02769|ALBU_BOVIN	498	235	2604.339	652.093	4	-4.7	19.53	contaminant
205	LDQELKLIGEYGLR-KTYVTPR-a6-b1	sp|P46781|RS9_HUMAN	sp|P46781|RS9_HUMAN	30	11	2647.45	662.87	4	-3.2	19.47	
206	AASVPKVETVK-VKAEPFIK-a6-b2	sp|P55010|IF5_HUMAN,db|PROT06|IF5_HUMA	sp|P55010|IF5_HUMAN,db|PROT06|IF5_HU	433	394	2196.269	550.075	4	-3.5	19.38	
207	VKAEPFIK-ELAKEIR-a2-b4	sp|P55010|IF5_HUMAN,db|PROT06|IF5_HUMA	sp|P55010|IF5_HUMAN,db|PROT06|IF5_HU	394	389	1926.112	643.045	3	-3.5	18.99	
208	YICDNQDTISSKLK-ECCDKPLLEK-a12-b5	sp|P02769|ALBU_BOVIN	sp|P02769|ALBU_BOVIN	297	304	3112.464	779.124	4	-4	18.25	contaminant
209	FQQKQIENVLR-TGFQAVTGKR-a4-b9	sp|P20042|IF2B_HUMAN	sp|P20042|IF2B_HUMAN	265	324	2603.41	651.86	4	-3.2	18.15	
210	AVLKFAAATGATPIAGR-EEQAAAEKAVTK-a4-b8	sp|P08865|RSSA_HUMAN	sp|P08865|RSSA_HUMAN	89	220	3025.627	757.415	4	-5.9	17.41	
211	GNKPWISLPR-ITPEEAKYK-a3-b7	sp|P62701|RS4X_HUMAN	sp|P62701|RS4X_HUMAN	233	120	2382.289	596.58	4	-2.4	17.06	
212	VKADRDESSPYAAMLAAQDVAQR-TKTPGPGAQSALR-a2-b2	sp|P62263|RS14_HUMAN	sp|P62263|RS14_HUMAN	63	106	3911.973	783.402	5	-1.9	16.02	
